# Structural basis of the substrate recognition and inhibition mechanism of *Plasmodium falciparum* nucleoside transporter PfENT1

**DOI:** 10.1038/s41467-023-37411-1

**Published:** 2023-03-28

**Authors:** Chen Wang, Leiye Yu, Jiying Zhang, Yanxia Zhou, Bo Sun, Qingjie Xiao, Minhua Zhang, Huayi Liu, Jinhong Li, Jialu Li, Yunzi Luo, Jie Xu, Zhong Lian, Jingwen Lin, Xiang Wang, Peng Zhang, Li Guo, Ruobing Ren, Dong Deng

**Affiliations:** 1 Department of Obstetrics, Key Laboratory of Birth Defects and Related Disease of Women and Children of MOE, State Key Laboratory of Biotherapy, West China Second Hospital, Sichuan University, Chengdu, 610041 China; 2 Shanghai Key Laboratory of Metabolic Remodeling and Health, Institute of Metabolism and Integrative Biology, Fudan University, Shanghai, 200438 China; 3 Warshal Institute of Computational Biology, School of Life and Health Sciences, the Chinese University of Hong Kong, Shenzhen, Guangdong 518172 China; 4 Shanghai Synchrotron Radiation Facility, Shanghai Advanced Research Institute, Chinese Academy of Sciences, Shanghai, 201204 China; 5 National Key Laboratory of Plant Molecular Genetics, Center for Excellence in Molecular Plant Sciences, Institute of Plant Physiology and Ecology, Chinese Academy of Sciences, Shanghai, 200032 China; 6 Frontier Science Center for Synthetic Biology and Key Laboratory of Systems Bioengineering of MOE, School of Chemical Engineering and Technology, Tianjin University, Tianjin, 300072 China; 7 State Key Laboratory of Biotherapy and Cancer Center, West China Hospital, Sichuan University, Chengdu, 610041 China; 8 Shanghai Qi Zhi Institute, Shanghai, 200030 China; 9 NHC key Laboratory of Chronobiology, Sichuan University, Chengdu, 610041 China; 10 Development and Related Diseases of Women and Children Key Laboratory of Sichuan Province, Sichuan University, Chengdu, 610041 China

**Keywords:** Cryoelectron microscopy, Cryoelectron microscopy, Permeation and transport

## Abstract

By lacking de novo purine biosynthesis enzymes, *Plasmodium falciparum* requires purine nucleoside uptake from host cells. The indispensable nucleoside transporter ENT1 of *P. falciparum* facilitates nucleoside uptake in the asexual blood stage. Specific inhibitors of PfENT1 prevent the proliferation of *P. falciparum* at submicromolar concentrations. However, the substrate recognition and inhibitory mechanism of PfENT1 are still elusive. Here, we report cryo-EM structures of PfENT1 in apo, inosine-bound, and inhibitor-bound states. Together with in vitro binding and uptake assays, we identify that inosine is the primary substrate of PfENT1 and that the inosine-binding site is located in the central cavity of PfENT1. The endofacial inhibitor GSK4 occupies the orthosteric site of PfENT1 and explores the allosteric site to block the conformational change of PfENT1. Furthermore, we propose a general “rocker switch” alternating access cycle for ENT transporters. Understanding the substrate recognition and inhibitory mechanisms of PfENT1 will greatly facilitate future efforts in the rational design of antimalarial drugs.

## Abstract (web summary)

PfENT1 is a promising antimalarial drug target. Here, authors report cryo-EM structures of PfENT1 that, together with biochemical work, suggests PfENT1 is an inosine transporter and describe the inhibitory mechanism of the endofacial inhibitor, GSK4.

## Introduction

Malaria, caused by infection with *Plasmodium* parasites, remains a severe infectious disease affecting human health worldwide. According to the World Health Organization, there were 241 million infection cases worldwide, and 627 000 people died from malaria in 2020^1^. Over the past several decades, many antimalarial drugs, including chloroquine and artemisinin, have been used to eradicate malaria^2,3^. However, the emergence drug-resistant *Plasmodium* parasites essentially delay malaria elimination^4–6^. As a result, there is a pressing need to develop antimalarial drugs^7–9^.

Malaria parasites, missing the de novo purine synthesis pathway, are purine auxotrophs^10,11^. Consequently, the salvage pathway mediated by nucleoside/nucleobase transporters is essential to maintaining DNA/RNA synthesis and proliferation of *Plasmodium* parasites^12–14^*. P. falciparum* nucleoside transporter 1 (PfENT1, encoded by *PF3D7_1347200)*, located predominantly in the parasite plasma membrane (PPM), is the primary nucleoside transporter mediating nucleoside uptake from erythrocytes^15–18^. Previous investigations have clarified that targeting the purine uptake pathway interferes with the survival of *Plasmodium*^19,20^. Crucially, PfENT1, a member of equilibrative nucleoside transporters (ENT, SLC29) family, shares only 17% sequence identity with the human equilibrative nucleoside transporter ENT1 (hENT1) and is unsusceptible to hENT1 inhibitors^16^, which provides an opportunity to design inhibitors targeting PfENT1 specifically. As expected, the reported PfENT1 inhibitors, including GSK4 (5-methyl-N-[2-(2-oxo-1-azepanyl)ethyl]−2-phenyl-1,3-oxazole-4-carbox-amide), disrupt the transport activity of PfENT1 and prevent the proliferation of parasites^21,22^. Hence, PfENT1 is a promising target to be exploited for novel antimalarial drug development^23–25^.

Here, we report three cryo-EM structures of PfENT1 in the apo, inosine-bound, and inhibitor-bound states at a resolution of 3.3 Å, 3.1 Å, and 4 Å, respectively. With extensive biochemical analysis, we decipher the inosine-binding site of PfENT1. Our structures also suggest the “rocker switch” transport model for ENTs^26^. Moreover, the structure of PfENT1 in complex with GSK4 provides a detailed inhibitory mechanism of the specific inhibitor. Our investigation will firmly deepen the understanding of the nucleoside transport mechanism and facilitate antimalarial drug design.

## Results

### Characterization of the *P. falciparum* nucleoside transporter PfENT1

**Fig. 1:**
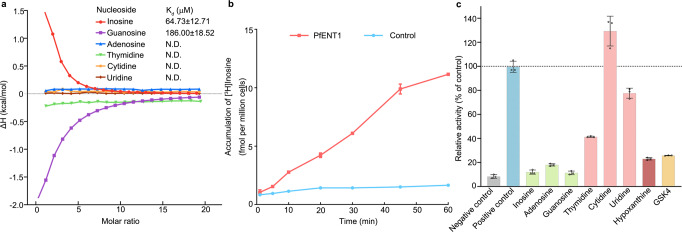
Characterization of PfENT1. **a** Nucleoside binding by PfENT1 as measured by ITC. A representative ITC experiment is presented. The binding affinity (K_d_) is presented as the value of the mean ± SD (*n* = 3), n means independent experiment. N.D., not detectable. Supplementary Fig. 2 shows the original ITC data and analyses. **b** The inosine transport activity of PfENT1. Time course for uptake of inosine in the knock-in yeast strain. The yeast strain without PfENT1 was tested as a control. Three independent experiments are performed for each point. Data are presented as mean ± SD. **c** Nucleoside competition and compound inhibition of PfENT1. The transport of [^3^H]-inosine was examined in uptake assays at 30 min in the presence of the indicated nucleosides/nucleobase (5 mM) and inhibitor GSK4 (10 μM). The yeast strain without PfENT1 was tested as a negative control. The PfENT1 knock-in yeast strain in the absence of non-radioactive nucleosides and inhibitor was test as positive control. Three independent experiments are performed for each substrate or inhibitor. Data are presented as mean ± SD. Source data are provided as a Source Data file.

Previous studies reported that PfENT1 has transport activity to various nucleosides and nucleobases^23,27,28^, yet the preferred substrate is controversial. Therefore, we overexpressed wild-type PfENT1 in *Sf9* insect cells, purified the monodisperse peak in solution (Supplementary Fig. 1), and measured the binding affinities between wild-type PfENT1 and various nucleosides via isothermal titration calorimetry (ITC) (Fig. 1a and Supplementary Fig. 2). As a result, PfENT1_WT_ binds to inosine and guanosine with binding affinities of approximately 64.3 μM and 186 μM, respectively (Fig. 1a and Supplementary Fig. 2). In contrast, the binding between PfENT1_WT_ and adenosine, as well as three pyrimidine nucleosides, including thymidine, cytidine, and uridine, was undetectable (Fig. 1a and Supplementary Fig. 2). Intriguingly, the inosine titration with PfENT1_WT_ is endothermic, whereas the guanosine titration is exothermic (Fig. 1a and Supplementary Fig. 2).

To further validate the inosine transport activity of PfENT1, we generated yeast strain deleted for the uridine transporter FUI1 then knocked in the codon-optimized PfENT1^21^. We performed a nucleoside uptake assay using the PfENT1 knock-in (KI) yeast strain (Fig. 1b). The^3^H-labeled inosine gradually accumulated in the KI strain over time but was not enriched in the control group (Fig. 1b). The competition assay was also conducted by using various nonradioactive nucleosides (Fig. 1c). The uptake of radioactive inosine was abolished by adding 5 mM nonradioactive inosine or guanosine (Fig. 1c). The inhibition efficiency of adenosine is relatively lower than that of inosine and guanosine (Fig. 1c). Pyrimidine nucleosides, such as thymidine and uridine, partially reduce inosine uptake. However, 5 mM cytidine did not influence inosine uptake (Fig. 1c). Interestingly, hypoxanthine, the base moiety of inosine, is known as a putative substrate of PfENT1^23^. Although the hypoxanthine-binding was undetectable, the partial inhibition by hypoxanthine is similar to that of purine nucleosides (Fig. 1c, Supplementary Fig. 2g), suggesting that hypoxanthine could also be imported. Recently, performing a high-throughput screen (HTS), researchers identified six potent PfENT1 inhibitors from GlaxoSmithKline’s compound library^22^. We synthesized one of the inhibitors, GSK4, and confirmed that 500 nM GSK4 effectively inhibited the transport activity of PfENT1 (Fig. 1c and Supplementary Fig. 3).

Considering inosine and hypoxanthine as the major purines in human blood^29–33^, we suggest that PfENT1 is an inosine transporter. Interestingly, LdNT2, the nucleoside transporter of another parasite, *Leishmania donovani*, was also identified as an inosine/guanosine transporter^34^. The purine nucleosides (inosine and guanosine) are the primary substrates of PfENT1, which is different from the human equilibrative nucleoside transporter (hENT1) with the specific recognition of the adenine base of nucleoside^35^.

### The overall structure of PfENT1

To obtain the structure of PfENT1, we performed comprehensive crystallization trials using engineered proteins and nanobody-bound transporter complexes. The PfENT1_Y190A_ mutant with a similar inosine-binding affinity presented better solution properties (Supplementary Fig. 1) and was used in the subsequent structural investigations.

**Fig. 2:**
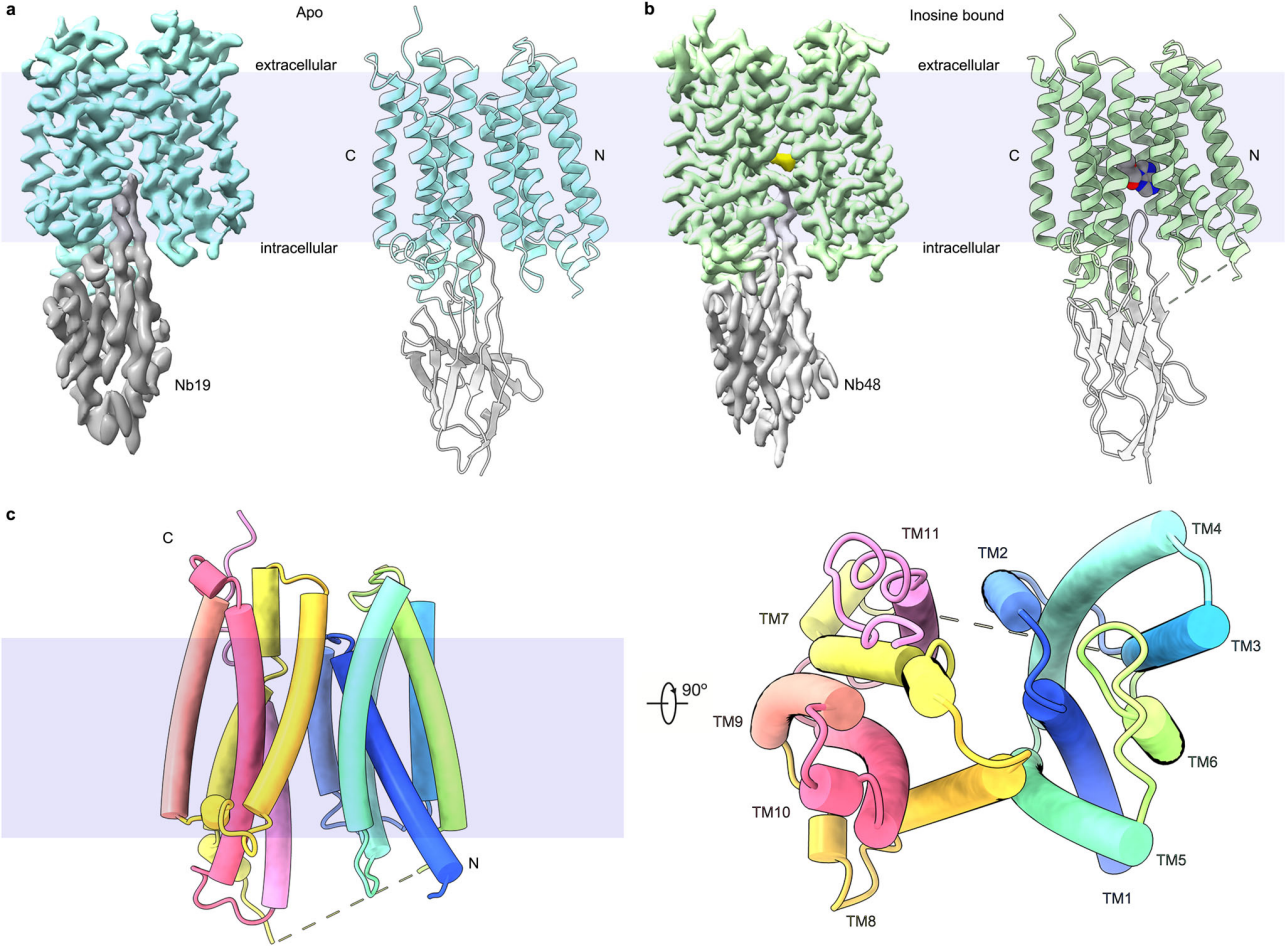
The overall structure of PfENT1. **a** Density map and structure of the PfENT1_Y190A_-Nb19 complex. The map and structure of PfENT1_Y190A_ are colored cyan, and the map and structure of Nb19 are shown in gray. **b** Density map and structure of the inosine-bound PfENT1_Y190A_-Nb48 complex. The map and structure of PfENT1_Y190A_ are colored green, and the map and overall structure of Nb48 are shown as light gray. The inosine molecule is shown in spheres. **c** The overall structure of PfENT1_Y190A_. The 11 TMs of PfENT1_Y190A_ are represented as rainbow-colored cylinders and viewed at two angles.

After extensive crystallization trials, we turned to cryo-EM to determine the structures of PfENT1. PfENT1 contains 422 residues without a predicted soluble domain, which is too small and a major challenge for cryo-EM analysis. Therefore, we generated two nanobodies, Nb19 and Nb48, to enlarge the particle size and create a soluble region (Supplementary Fig. 1c)^36^. Although the molecular weights of the two PfENT1-Nb complexes are less than 70 kDa, we obtained complex structures of PfENT1_Y190A_-Nb19 (apo) and PfENT1_Y190A_-Nb48 (inosine-bound) at 3.3 Å and 3.1 Å, respectively (Fig. 2a, b, Supplementary Fig. 4 and Supplementary Table 1). In particular, the resolution of the transmembrane domain of PfENT1_Y190A_-Nb48 reaches approximately 2.6 Å. The visibly clear density simplified the subsequent building of an accurate model of PfENT1 with residues 30-211 and 228-406 (Fig. 2, Supplementary Fig. 4 and Supplementary Fig. 5). Although Nb19 and Nb48 shared low sequence similarity of complementarity determining regions (CDRs), they bind to PfENT1 with a similar pattern (Supplementary Fig. 6). CDR loops 1 and 2 of both nanobodies interact with the intracellular surface of the carboxyl domain of PfENT1. In contrast, the third CDR loops insert into the central cavities of PfENT1. Intriguingly, Arg107 in the CDR3 loop of Nb19 interacts with Phe139 of PfENT1, blocking the transport pathway (Supplementary Fig. 6). Therefore, two nanobodies lock and stabilize PfENT1 in the inward open state.

The overall structure of PfENT1 contains eleven transmembrane (TM) helixes with pseudosymmetry (Fig. 2c). TM1-6 and TM7-11 form two bundles, which we defined as the amino (N) domain and carboxyl (C) domain, respectively (Fig. 2b). This structural architecture is quite similar to the first structure of ENT family-hENT1^37^, despite PfENT1 only sharing ~17% sequence identity with hENT1 (Supplementary Fig. 7). A previous study speculated that ENT transporters belong to the major facilitate superfamily (MFS), a typical twelve transmembrane helix transporter family with pseudosymmetrical amino (TM1-6) and carboxyl (TM7-12) domains^38,39^. Similar structural features confirm the hypothesis of the evolutionary relationship of MFS-type and ENT-type nucleoside transporters^40^, indicating that the two types of transporters share a similar transport mechanism.

### The nucleoside binding site of PfENT1

**Fig. 3:**
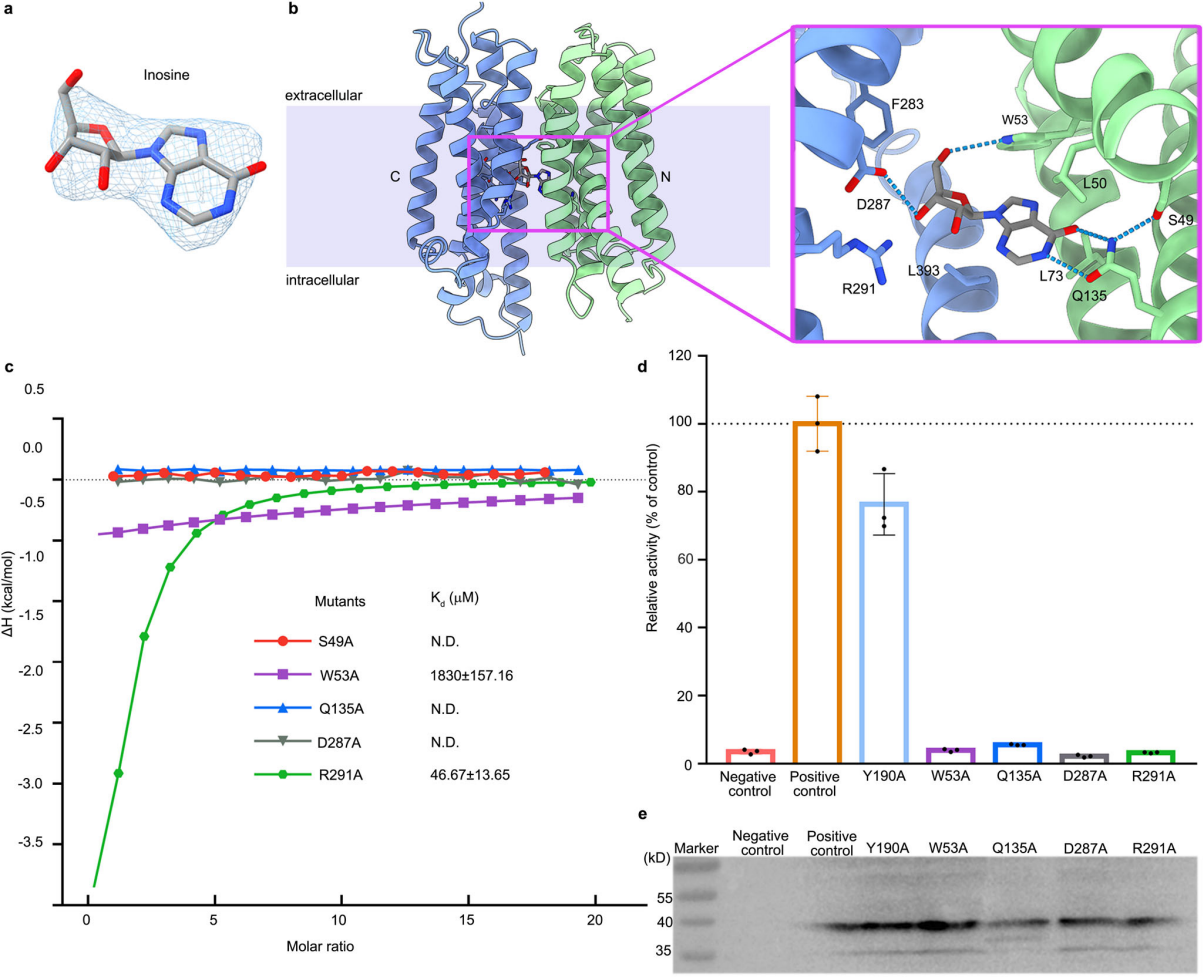
Substrate binding site of PfENT1. **a** Density map of inosine in the cavity of PfENT1 (6 σ, blue mesh). The density is fitted to the inosine molecule (sticks). **b** Inosine binding site of PfENT1. Dashed lines denote the inosine coordination in PfENT1. **c** Inosine binding affinity of the PfENT1 mutant (S49A, W53A, Q135, D287A, R291A) measured by ITC. The binding affinity (K_d_) is presented as the value of the mean ± SD (*n* = 3), n means independent experiment. N.D., not detectable. Supplementary Fig. 8 shows further ITC data and analyses. **d** Inosine uptake assay of PfENT1 mutants. Three independent experiments are performed for each construct. Data are presented as mean ± SD (*n* = 3). **e** Expression of the codon-optimized PfENT1-HA proteins in an *fui1*Δ S. cerevisiae strain were detected by western blot probed with anti-HA antibodies. Source data are provided as a Source Data file.

To decipher the substrate recognition mechanism, we determined the structure of the PfENT1_Y190A_-Nb48 complex in the presence of inosine. Comparing the density of PfENT_Y190A_-Nb19, the omitted density indicative of one inosine molecule was clearly visible in the central cavity of the PfENT1_Y190A_-Nb48 structure (Fig. 3a). The base and ribose moieties of inosine point to the N and C domains, respectively, which is similar with the adenosine analog-NBMPR binding site of hENT1^37^(Fig. 3b and Supplementary Fig 8)^37^. Notably, the ketone group of inosine, which also exists in guanosine, forms a hydrogen bond with the amino group of Gln135 on TM4 of PfENT1 (Fig. 3b). The interaction is possibly compromised by substituting the ketone group with an amino group in adenosine, consistent with our biochemical data (Fig. 1a). Additionally, the rotamer of Gln135 is stabilized by interacting with Ser49 (Fig. 3b), which is critical for inosine binding. The results showed complete abolishment of the inosine binding of the S49A and Q135A mutants (Fig. 3c and Supplementary Fig.8). Furthermore, the hydrophobic residues Leu50, Trp53, and Leu73 limit the rotation of inosine (Fig. 3b). Considering the conservation of Leu50 and Leu73 in human and *Plasmodium* ENT1s, these two residues may also contribute to the substrate specificity of PfENT1 similar to that in human ENT1^37^. The ribose moiety of inosine forms direct contact with Trp53, Asp287, and Arg291 (Fig. 3b). Mutant W53A showed 30-fold weaker binding than the wild type, and D287A completely lost inosine binding (Fig. 3c and Supplementary Fig. 8). However, another mutant, R291A, maintains inosine binding, indicating a weak interaction between Arg291 and inosine (Fig. 3c and Supplementary Fig. 8). The results of inosine uptake assays further confirmed the crucial roles of these residues (Fig. 3d). PfENT_Y190A_, used for structural determination, maintained approximately 70% transport activity compared to PfENT_WT_. W53A, Q135A, and D287A abolished the transport activity of PfENT1. Interestingly, the mutant R291A maintained the binding capability but lost the transport activity of inosine (Fig. 3d). As reported, Arg291 is essential for the transport activity of ENTs^37,41,42^. Arg345 in hENT1, the corresponding residue of Arg291 of PfENT1, is involved in adenosine analog binding in the outward-facing conformation^37^. The mutant R291A lost the transport activity of PfENT1, suggesting that Arg291 may play an important role in the alternating access cycle (Fig. 3c and Supplementary Fig. 8). The Consurf evolutionary conservation analysis showed that all mentioned residues above, except Ser49 and Leu73, are highly conserved (Supplementary Fig. 9). In addition, several hydrophobic residues, including Phe283 and Leu393, are involved in nucleoside recognition. Interestingly, none of the residues of Nb48 have direct polar interaction with inosine, except for the potential hydrophobic interaction between Try104 and the ribose group of inosine (Supplementary Fig. 6b).

Intriguingly, the bacterial MFS nucleoside transporter (NupG) and concentrative nucleoside transporters (CNTs) recognize the ribose moiety of nucleoside via extensive polar contacts^43–45^, whereas there is only one polar residue (Asp287) from the C domain of PfENT1 involved in ribose recognition directly. The lower availability of residues interacting with the ribose group is probably responsible for the nucleobase transport activity and substrate specificity of PfENT1^37,41^. In addition, we found four conserved polar residues (Asn250, Thr253, Asn354, and Asn358) from the C domain, which are all within 6 Å of the ribose group of inosine (Supplementary Fig. 9).

### Alternating access cycle of ENTs

**Fig. 4:**
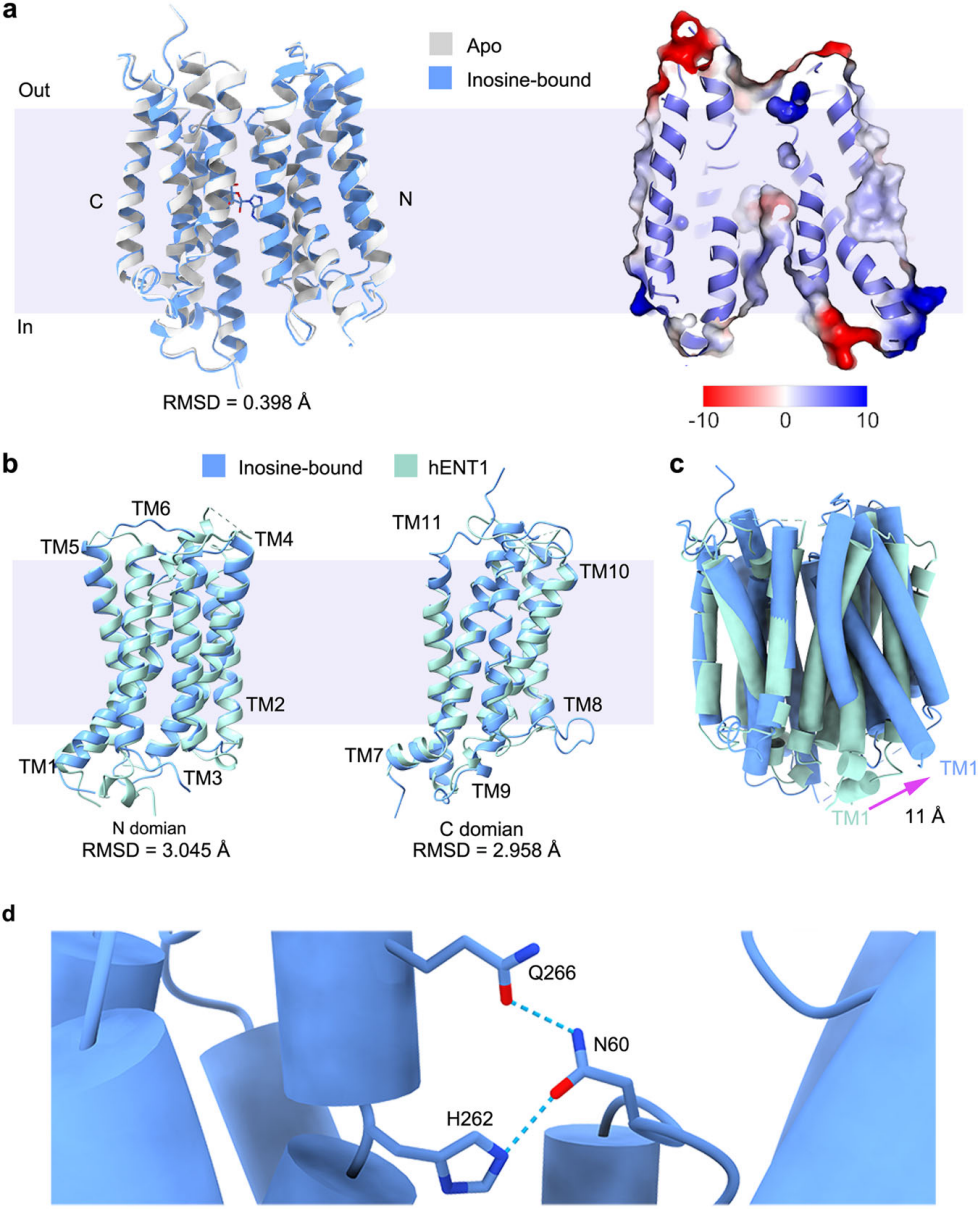
Alternating access cycle of ENTs. **a** Superposition of the apo PfENT1 and inosine-bound PfENT1 complex. The apo- and inosine-bound PfENT1 is shown as a cartoon with the colors of light gray and blue, respectively. Inosine is represented as sticks. **b** Superposition of the N and C domains of PfENT1 and hENT1. PfENT1 and hENT1 is shown as cartoon with the colors of blue and green, respectively. **c** Superposition of the PfENT1, and hENT1 overall structure. PfENT1 and hENT1 is shown as cartoon with the colors of blue and green, respectively. **d** Critical residues in the alternating access cycle. The key residues are shown as sticks, interaction between key residues is shown by dashed lines.

The two structures of PfENT1 present identical inward open conformations with r.m.s.d. value at 0.398 Å (Fig. 4a). Intriguingly, the structures of hENT1 were captured at the outward facing conformation^37^. The structural alignments between the individual amino or carboxyl domains of PfENT1 to hENT1 are similar to the r.m.s.d. value at approximately 3 Å (Fig. 4b). Notably, the amino domain of PfENT1 is deflected by approximately 11 Å when we superimposed the carboxyl domain of the PfENT1 and hENT1 (Fig. 4c). A predicted structure of PfENT1 on the alpha-fold protein structure database (https://www.alphafold.ebi.ac.uk/entry/Q8IDM6) shares the similar conformation with hENT1 (Supplementary Fig. 10a). The conformational change also is clearly shown when we superimposed the solved structures and the predicted model of PfENT1 (Supplementary Fig. 10).

The structures in inward open conformations give us valuable information to analyze the critical residues involved in conformational stabilization. In the inward-open state of PfENT1, the occluded extracellular gate is stabilized by extensive polar interactions. Asn60, His262, and Gln266 forming a hydrogen-bond network are conserved in plasmodium ENTs (Fig. 4d and Supplementary Fig. 6).

Consequently, PfENT1 probably undergoes rigid-body movement to alternate access for the receiving or releasing nucleoside molecule. The structural similarity of PfENT1 and hENT1 also suggests a consensus rigid-body movement transport mechanism of ENT transporters, similar to the classic “rocker switch” model of MFS transporters^26,46–48^.

### Inhibition mechanism of GSK4

**Fig. 5:**
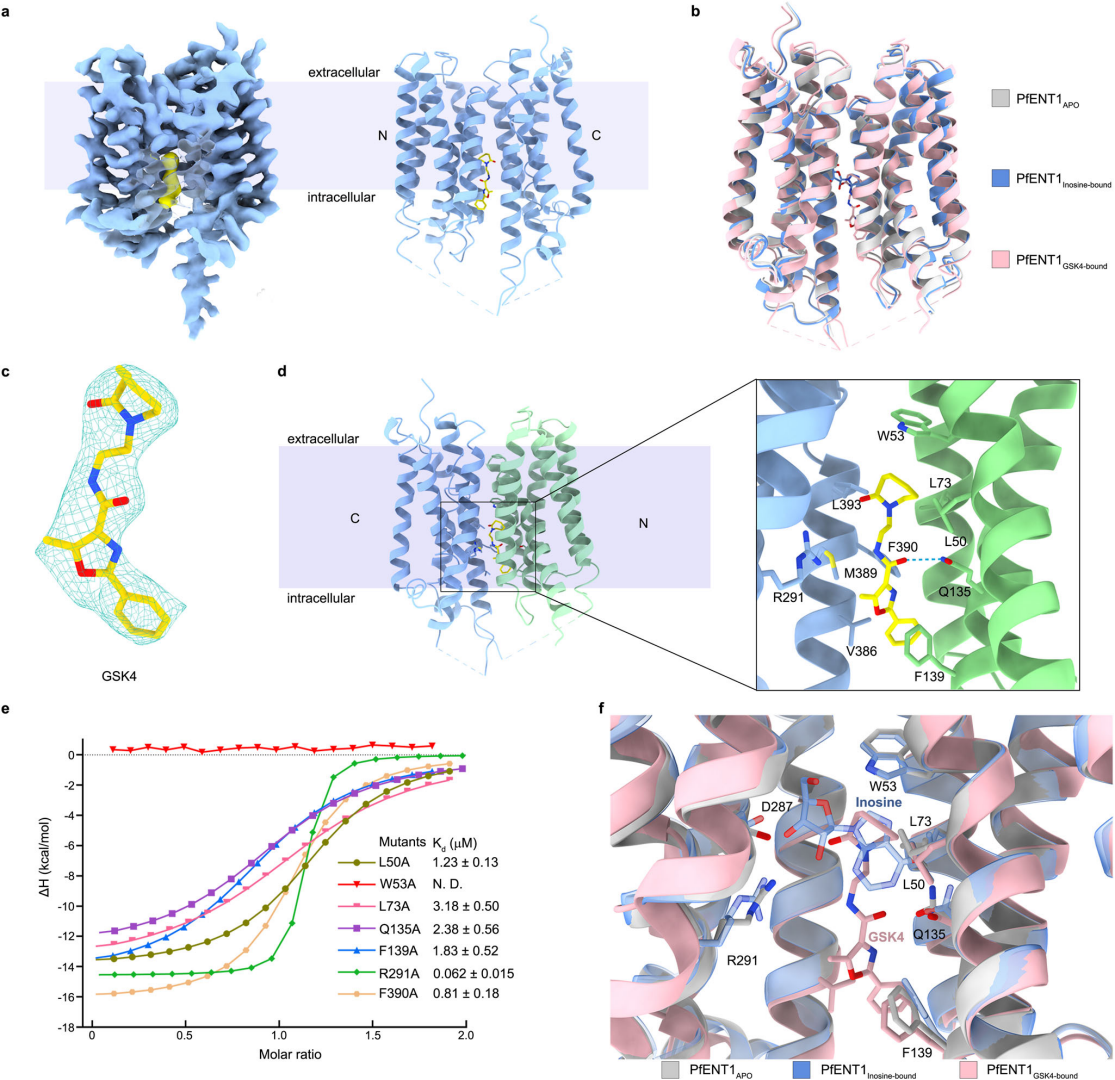
Inhibition mechanism of GSK4. **a** Density map and structure of PfENT1_GFP_. The map and structure of PfENT1 are colored blue. The map and structure of GSK4 are shown in yellow. **b** Superposition of the PfENT1-Apo, inosine-bound PfENT1 complex, and GSK4-bound PfENT1 complex. PfENT1-Apo, inosine-bound PfENT1 complex, and GSK4-bound PfENT1 complex are shown as cartoons with colors of gray, blue, and pink, respectively. **c** Density map of GSK4 in the cavity of PfENT1 (6 σ, blue mesh). The density is fitted to the GSK4 molecule (sticks). **d** GSK4 binding site of PfENT1. Dashed lines denote GSK4 coordination in PfENT1. **e** Inosine binding affinity of the PfENT1 mutant (W53A, Q135, F139, R291A) measured by ITC. The binding affinity (Kd) is presented as the value of the mean ± SD (*n* = 3), n means independent experiment. N.D., not detectable. Supplementary Fig. 13 shows the original ITC data and analyses. Source data are provided as a Source Data file. **f** Superposition of the PfENT1-Apo, inosine-bound PfENT1 complex, and GSK4-bound PfENT1 complex, PfENT1-Apo, inosine-bound PfENT1 complex, and GSK4-bound PfENT1 complex are shown as cartoons with colors of gray, blue, and pink, respectively. Key residues are shown as sticks. Inosine and GSK4 are shown as sticks with blue and pink colors, respectively.

To decipher the inhibitory mechanism of GSK4, we engineered PfENT1 with internal insertion of GFP between TM10 and TM11, termed PfENT1_GFP_ (Fig. 5a). PfENT1_GFP_ maintains inosine binding with a similar affinity as PfENT1_WT_ and PfENT1_Y190A_ (Fig. 1 and Supplementary Fig. 11). More importantly, GSK4 binds to PfENT1_WT_, PfENT1_Y190A_, and PfENT1_GFP_ with the same Kd value range of ~40 nM (Supplementary Fig. 11). Furthermore, we determined the structure of PfENT1_GFP_ in complex with GSK4 at 4.0 Å (Fig. 5a, Supplementary Fig. 12 and Supplementary Table 1). The overall structure of PfENT1_GFP_ presents a similar conformation as the structures of PfENT1_APO_ and PfENT1_inosine-bound_ (Fig. 5b).

An extra density appears in the central cavity, fitting with the GSK4 molecule well (Fig. 5c). GSK4 interacts with PfENT1 via polar contact between the carboxamide group of GSK4 and Gln135 (Fig. 5d). Q135A of PfENT1 dramatically reduced the binding affinity of GSK4 (Fig. 5e and Supplementary Fig. 13). Trp53, a conserved aromatic residue from ENTs, forms a CH-π interaction with the 2-oxo-1-azepanyl group of GSK4 (Fig. 5d). The W53A mutant abolished the GSK4 binding of PfENT1 (Fig. 5e and Supplementary Fig. 13). The benzene ring of GSK4 interacts with Phe139 via hydrophobic interaction (Fig. 5d). The mutant F139A reduces GSK4 binding (Fig. 5e and Supplementary Fig. 13). Interestingly, six identified inhibitors (GSK1-6) contain a conserved carboxamide group and distinct groups with cyclic structures^22^, suggesting that the polar contact with Gln135 is essential for compound recognition. The inhibitors with diverse cyclic groups may also share similar interactions with the aromatic residues. In addition, several hydrophobic residues, including Leu50, Leu 70 from the N domain, and Val386, Met389, Phe390, and Leu393 from the C domain, are involved in GSK4 recognition (Fig. 5d). The mutants L50A, L73A, and F390A also reduce GSK4 binding (Fig. 5e). Compared with the inosine-bound structure, GSK4 partially overlapped with the base moiety of inosine (Fig. 5f). Moreover, GSK4 occupies the nucleoside release tunnel of PfENT1 (Fig. 5f). Therefore, GSK4 is an endofacial inhibitor blocking the alternating access of PfENT1. However, due to the resolution limit of the GSK4 bound PfENT1 data, we cannot rule out other binding poses of GSK4 as shown in Supplementary Fig. 14.

## Discussion

Based on three structures of PfENT1 in the inward-open conformation and the outward-facing structure of hENT1, we proposed a general transport mechanism (Supplementary Fig. 15). In an alternating access cycle, PfENT1 completes nucleoside translocation via rigid-body movement of the N and C domains. Upon nucleoside binding into the central cavity of PfENT1, polar contacts form and trigger conformational changes. Although ENT transporters may undergo a similar rigid-body movement cycle due to the similar structural fold and conserved substrate recognition pattern of MFS^49^, the remaining question is whether the local conformational differences determine the binding kinetics and dynamics of various substrates, such as glucose transport by GLUTs^50^.

Targeting transporters and channels of *Plasmodium* parasites to eliminate malaria has attracted much attention from researchers^25,51^. Here, we report the structure of PfENT1 in complex with the antimalarial compound, which uncovers the inhibition mechanism of the small molecule. Most importantly, GSK4 forms one hydrogen bound with Gln135 from the N domain, suggesting that we can further optimize the existing inhibitors via potential interactions with polar residues from the C domain in the central cavity (Supplementary Fig. 10). Therefore, our study provides indispensable information for the rational design of antimalarial compounds.

## Methods

### Protein expression and purification

The codon-optimized cDNA of *P. falciparum* equilibrative nucleoside transporter 1 (PfENT1, UniProt No. Q9NIH7) was synthesized by GENEWIZ. For PfENT1_GFP_, the cDNA of GFP was cloned into PfENT1 between K370 and K371. All constructs were subcloned into a modified pFastBac1 with an N-terminal 10 X His tag. The baculovirus was generated in DH10Bac for recombinant protein expression. After 72 h of infection, Sf9 insect cells were harvested. For structural determination of the PfENT1 protein, Sf9 cells were solubilized in lysis buffer (25 mM Tris-HCl pH 8.0, 150 mM NaCl), 1% (w/v) lauryl maltose neopentyl glycol (LMNG, Anatrace), and protease inhibitor cocktail (0.8 μM aprotinin, 2 μM pepstatin, and 5 μg/ml leupeptin) at 4 °C for 2 h. After centrifugation at 20,000 × *g* for 60 min at 4 °C, the detergent-soluble supernatant was collected via centrifugation at 4 °C for 60 min and incubated with Ni-NTA resin (QIAGEN) at 4 °C for 30 min. The resin was further washed with 25 mM Tris-HCl pH 8.0, 150 mM NaCl, 10 mM imidazole, and 0.06% (w/v) glyc-diosgenin (GDN, anatrace) and eluted with buffer containing 25 mM Tris-HCl pH 8.0, 150 mM NaCl, 250 mM imidazole, and 0.06% (w/v) GDN.

The nanobodies for PfENT1 were generated by Chengdu NB Biolab Co., Ltd. Subsequently, the cDNA of nanobodies was subcloned into pET21b and overexpressed in *E. coli* BL21(DE3) cells. For recombinant expression, the cells were cultured at 37 °C and induced by 0.2 mM isopropyl β-D-thiogalactoside (IPTG) at 22 °C overnight once the cell density reached an OD_600_ of 0.8. The cells were harvested and homogenized in lysis buffer (25 mM Tris-HCl pH 8.0, 150 mM NaCl). The cells were lysed by a high-pressure homogenizer (ATS) and clarified via centrifugation. The supernatant was applied to Ni-NTA resin (QIAGEN) and eluted from the resin using buffer containing 25 mM Tris-HCl (pH 8.0), 30 mM NaCl, and 300 mM imidazole. The elution was further applied to ion-exchange chromatography (Source 15Q, GE Healthcare) and gel filtration (Superdex 200 Increase 10/300; GE Healthcare). Finally, nanobodies were eluted in buffer containing 25 mM Tris-HCl (pH 8.0) and 30 mM NaCl and concentrated to ~50 mg/ml for further purification.

The purified PfENT1 proteins were concentrated and incubated with nanobody at a molar ratio of 1:1.5 at 4 °C for 1 h. The PfENT1-nanobody complex was subjected to size-exclusion chromatography (Superose 6 increase 10/300; GE Healthcare) in buffer containing 25 mM MES pH 6.0, 150 mM NaCl, and 0.06% (w/v) GDN. The peak fractions were pooled and concentrated to 8-12 mg/mL for cryo-EM analysis.

For structural determination of PfENT1_GFP_ protein, Sf9 cells (Thermofisher, #11496015) were solubilized in lysis buffer (25 mM Tris-HCl pH 8.0, 150 mM NaCl), 2% (w/v) n-dodecyl-β-D-maltopyranoside (DDM), and protease inhibitors (0.8 μM aprotinin, 2 μM pepstatin, and 5 μg/ml leupeptin) at 4 °C for 1.5 h. After centrifugation at 18,000 × *g* for 30 min at 4 °C, the detergent-soluble supernatant was collected and incubated with Ni-NTA resin (QIAGEN) at 4 °C°C for 30 min. The resin was further washed with 25 mM Tris-HCl pH 8.0, 150 mM NaCl, 30 mM imidazole, and 0.02% (w/v) n-dodecyl-β-D-maltopyranoside (DDM, Anatrace) and eluted with buffer containing 25 mM Tris-HCl pH 8.0, 150 mM NaCl, 300 mM imidazole, and 0.02% (w/v) n-dodecyl-β-D-maltopyranoside (DDM, Anatrace). The purified protein was concentrated and subjected to size-exclusion chromatography (Superdex 200 Increase 10/300; GE Healthcare) in buffer containing 25 mM Tris-HCl pH 8.0, 150 mM NaCl, and 0.02% (w/v) n-dodecyl-β-D-maltopyranoside (DDM, Anatrace). The peak fractions were pooled and concentrated to 4-12 mg/mL for cryo-EM analysis.

For the in vitro binding assay, Sf9 cells were solubilized in lysis buffer containing 2% (w/v) n-dodecyl-β-D-maltopyranoside (DDM, Anatrace) and protease inhibitor cocktail (0.8 μM aprotinin, 2 μM pepstatin, and 5 μg/ml leupeptin) at 4 °C for 2 h. The soluble supernatant was collected via centrifugation and incubated with Ni-NTA resin (QIAGEN) at 4 °C for 1 h. The resin was washed and eluted using lysis buffer containing 0.05% (w/v) DDM and 30 mM/300 mM imidazole. The protein was further purified by size-exclusion chromatography (Superdex 200 Increase 10/300; GE Healthcare) in buffer containing 25 mM MES (pH 6.0), 150 mM NaCl, and 0.05% (w/v) DDM. The peak fractions were pooled and concentrated to 10~50 μM for the nucleoside-binding assay.

### GSK4 synthesis procedure

The synthesis process is summarized in Supplementary Fig. 3. Into a two-neck round-bottomed flask equipped with a mechanical stirrer and a dropping funnel. To a stirred solution of aryl acid in CH_2_Cl_2_ at room temperature, oxalyl chloride (2.0 equiv) and 2 drops of DMF were added, and the mixture was stirred at room temperature overnight. The resulting mixture was concentrated under reduced pressure to quantitatively afford acid chloride **1**.

6-Bromohexanoic acyl chloride **3** was stirred with BocNH(CH_2_)_2_NH_2_ **2** (1.0 equiv) and K_2_CO_3_ (1.5 equiv) in CH_2_Cl_2_ at 0 °C for 30 min and 25 °C overnight. The sample was filtered to remove solids and washed with water, 1 M HCl, and brine. The residue was concentrated under reduced pressure to afford pale yellow crystals **4**.

A suspension of sodium hydride in oil (40% w/w, 3.5 equiv.) was suspended in dry THF, and a solution of **4** (1.0 equiv.) in dry THF was added dropwise at rt over 30 minutes. The mixture was then stirred for an additional 12 h. The mixture was then cooled (ice), acidified (4 M HCl), and extracted with DCM. The combined organic extracts were washed with brine, dried, concentrated in vacuo, and purified by column chromatography to yield compound **5**.

TFA (1.5 mL) was added to a solution of compound **5** (2.0 mmol, 1.0 equiv) in 5 mL DCM. The solution was stirred at room temperature for 2-3 h. The reaction was completely detected by TLC. The reaction mixture was quenched by the addition of 2 M NaOH and extracted with ethyl acetate. The combined organic layers were washed with brine, dried over Na_2_SO_4_, filtered, and concentrated under reduced pressure to give a residue. The residue was purified by column chromatography in ethyl acetate/petroleum to give desired compound **6**.

Compound **6** (1.05 equiv.) and triethylamine (2.0 equiv.) were combined in dichloromethane under nitrogen, giving a colorless suspension. Benzoyl chloride **1** (1.0 equiv.) in dichloromethane was added dropwise over 10 min, and the solution was stirred overnight. The reaction mixture was quenched by the addition of water, and the mixture was extracted with CH_2_Cl_2_. The combined organic layers were dried over Na_2_SO_4_, filtered, and concentrated under reduced pressure to give a residue. The residue was purified by column chromatography in ethyl acetate/petroleum to give desired compound **7**.

### NMR

10 mg GSK-4 compound dissolved in 0.5 mL CDCl3 and tested for 1H NMR spectra on an AMX 400 NMR spectrometer from Bruker, Germany. 1H NMR was reported as follows: chemical shift, multiplicity (s = singlet, d = doublet, t = triplet, q = quadruplet, m = multiplet), coupling constant (J values) in Hz and integration. Chemical shifts (δ) were reported with respect to the corresponding solvent residual peak at 7.26 ppm for CDCl3 for 1H NMR.

### Isothermal titration calorimetry (ITC)

The binding affinity between wild-type and mutant PfENT1 with nucleosides and GSK4 was measured using a Micro-Cal ITC200 (Malvern). All proteins were prepared in 25 mM MES (pH 6.0), 150 mM NaCl, and 0.05% (w/v) DDM. Nucleosides (5~10 mM) and GSK4 (100-200 μM) dissolved in the same buffer were titrated against 20–50 μM PfENT1 proteins at 25 °C. PEAQ-ITC analysis software (MicroCal) was employed to analyze the data. All titrations were repeated three times.

### PfENT1-HA tagging in *S. cerevisiae*

The *S. cerevisiae* strain (ATCC, #201388) BY4741 (*MATa his3Δ1 leu2Δ0 met15Δ0 ura3Δ0*) was used as the starting yeast strain. Yeast strains were cultured in YPD medium (20 g/L peptone, 10 g/L yeast extract, and 20 g/L dextrose). The antibiotic G418 was supplemented at a concentration of 250 μg/mL to select strains carrying the KanMX marker. *E. coli* DH5α was used for subcloning.

Codon-optimized PfENT1 (for expression in yeast) was synthesized by Sangon Biotech. The carboxyl terminus of PfENT1 was HA-tagged through PCR. The TEF1p promoter and PGK1t terminator were amplified from BY4741 genomic DNA. Overlap PCR was applied to generate the gene overexpression cassette of PfENT1 (TEF1p promoter-PfENT1-PGK1t terminator). The two 600-bp homologous arms corresponding to the upstream and downstream regions of the *FUI1* gene were amplified from BY4741 genomic DNA. The gene cassette of the ‘TEF1p promoter-PfENT1-PGK1t terminator’ together with the two homologous arms was cloned into the PUC19 plasmid through Gibson assembly^52^, generating the final plasmid PUC19: PfENT1. PfENT1 mutations were generated using point mutation and verified by Sanger sequencing. The spacer targeting the *FUI1* gene (5ʹ-ACTCGAAGTTCGTCAAATTG-3ʹ) was cloned into the pCas-BsaI plasmid to generate the pCas-FUI1 plasmid as described previously^53^.

To obtain *fui1Δ* and PfENT1 (or its mutations) overexpression in the S. cerevisiae strain, 1 μg pCas-FUI1 plasmid and 15 μg PUC19:PfENT1 were transformed into yeast using the LiAc/ssDNA/PEG method^54^. Then, the strains were plated on a YPD plate supplemented with G418. Clones were screened using PCR and finally verified through Sanger sequencing.

### Western blotting

Approximately 2 × 10^8^ cells in mid-log phase growth were harvested by centrifugation at 3000 × *g* for 8 min at 4 °C. Pellets were washed with 2 ml lysis buffer (25 mM Tris 8.0 150 mM NaCl) and resuspended in 20% Snailase (v/v) with reaction buffer overnight at 37 °C. The digested yeast was harvested with 100 μl lysis buffer and prepare samples. For western blots, the following parameters were used: wet transfer (25 mM Tris 190 mM glycine 20% [v/v] methanol in transfer buffer) for 90 min / 100 V / 4 °C, PVDF membrane (0.22 μm). Blocking was applied for 2 h at room temperature using 5% (w/v) non-fat dry milk in TBST (25 mM Tris-HCl pH 7.5, 150 mM NaCl, 0.05% Tween 20). Primary antibodies (rabbit anti HA 1:4000, AbClonal #AE036) were applied overnight at 4 °C in 5% (w/v) non-fat dry milk in TBST. Secondary antibodies (goat anti-mouse HRP 1:4000, Zenbio, #511203) were applied for 1.5 h at room temperature using 5% (w/v) non-fat dry milk in TBST.

### Inosine uptake assay

Cells were grown overnight in YPD medium to mid-log phase and harvested by centrifugation at 3000 × *g* for 5 min. Cells were washed three times in 2% glucose. The yeast pellets were resuspended in uptake assay buffer (25 mM Tris-HCl 8.0,150 mM NaCl, 2% glucose) to a final concentration of 2 × 10^8^ cells mL^–1^. The uptake assay was initiated by combining 100 μL of prewarmed (30 °C) cells and 100 μL uptake assay buffer mixture: 25 mM Tris-HCl 8.0, 150 mM NaCl, 2% glucose, 200 nM [^3^H] Inosine ([8-^3^H] inosine; 20 Ci mmol^–1^, American Radiolabeled Chemicals). Cells were removed onto a membrane filter at the appropriate times to generate the time course. After washing by vacuum filtration with 2 ml ice-cold 2% glucose, the radioactivity retained on the filter was incubated with 1 mL of ULTIMA GOLD scintillation fluid (Perkin Elmer, 77-15311). Counts were measured using a liquid scintillation analyzer (Perkin Elmer, Tri-Carb 5110TR).

For competition assays, the indicated nucleosides and nucleobase were added into the external uptake assay buffer (25 mM Tris-HCl 8.0, 150 mM NaCl, and 2% glucose) to a final concentration of 5 mM, and the transport reaction was stopped at 30 min. The reading of the competition assays was normalized against the one without a competitor, which had the uptake of radiolabeled inosine set as 100%. All uptake assays were performed at 30 °C and repeated three times. Error bars represent the s.d. The final result was analyzed in the GraphPad Prism 8 software (version 7.0.0).

### Cryo-EM sample preparation and data acquisition

A total of 3.5 μL of the concentrated complex at approximately 8-12 mg/mL (9.4, 10.9, 11.8 mg/mL for the PfENT1_Y190A_-Nb48 complex and 8.0 mg/mL for the PfENT1_Y190A_-Nb19 complex) was applied to glow-discharged, holey carbon-coated grids (Quantifoil 300 mesh, Au R1.2/1.3). The grids were blotted for 3 s and flash-frozen in liquid ethane using a Vitrobot (Mark IV, Thermo Fisher Scientific). Images were recorded on a 300 kV Titan Krios G3i electron microscope (Thermo Fisher Scientific) equipped with a Gatan K3 Summit direct detector and a GIF Quantum energy filter (slit width 20 eV). Movie stacks were collected using SerialEM^55^ in counting mode at a magnification of 105,000x with a corresponding pixel size of 0.83 Å. Each movie stack of PfENT1_Y190A_-Nb19 complex with 50 frames was exposed for 3 s at a dose rate of 13.2 e/px/s, resulting in a total dose of 57.5 per Å^2^ over 50 frames. The defocus range was set from −1.2 to −1.8 μm. Each movie stack of the PfENT1_Y190A_-Nb48-inosine complex was exposed for 2.5 s at a dose rate of approximately 15.5 e/px/s, resulting in a total dose of approximately 56.3 per Å^2^ over 50 frames. The defocus range was set from −1.0 to −1.8 μm. A total of 4631 movie stacks were collected for the PfENT1_Y190A_-Nb19 complex. A total of 6916 movie stacks were collected for the PfENT1_Y190A_-Nb48 complex.

For the PfENT1_GFP_ sample_,_ 3.5 μL of the concentrated PfENT1_GFP_-GSK4 complex, approximately 4.0 mg/mL, was applied to glow-discharged holey carbon-coated grids (Quantifoil 300 mesh, Au R1.2/1.3). The grids were blotted for 2.5 s and flash-frozen in liquid ethane using a Vitrobot (Mark IV, Thermo Fisher Scientific). Images were recorded on a 300 kV Titan Krios G3i electron microscope (Thermo Fisher Scientific) equipped with a Gatan K2 Summit direct detector and a GIF Quantum energy filter (slit width 20 eV). Movie stacks were collected using EPU in counting mode at a magnification of 130,000x with a corresponding pixel size of 1.1 Å. Each movie stack of the PfENT1_GFP_-GSK4 complex with 30 frames was exposed for 6 s at a dose rate of 6.463 e/px/s, resulting in a total dose of 52.452 per Å^2^ over 30 frames. The defocus range was set from −1.1 μm to −1.8 μm. A total of 9044 movie stacks were collected for the PfENT1_GFP_-GSK4 complex.

### Data processing

Movies frames were aligned using MotionCor2^56^ with 5 by 5 patches. Micrograph contrast transfer function (CTF) estimations were performed by CTFFind4^57^ using micrographs without dose-weighting. The dose-weighted micrographs were used for particle picking and further processing. Particles were automatically picked by Gautomatch. For data of the PfENT1_Y190A_-Nb48-inosine complex, 4,651,677 particles were picked. Micrographs with estimated Ctf Max Resolution (> 5 Å) and Ctf Figure Of Merit (> 0.3) were selected for further processing. A total of 6778 micrographs were selected with 4,572,619 particles. Particles were extracted by Relion3.1^58^ with a box size of 72 pixels and a pixel size of 3.32 Å. Iterative 2D classifications were performed using cryoSPARC v.2.14.2^59^. A total of 1,129,242 particles were selected and then recentered and re-extracted by Relion with a box size of 144 pixels and a pixel size of 1.66 Å. Three parallel ab initio reconstructions were performed in cryoSPARC. A total of 854,399 particles from combined good classes were selected. These particles were subjected to one round of heterogeneous refinement, and 492,596 particles were selected. Next, particles were subjected to one round of nonuniform refinement, yielding a 3.8 Å map. Three rounds of heterogeneous refinement were performed, and 243,894 particles were selected. One round of nonuniform refinement was performed, yielding a 3.4 Å map. Particles were recentered and re-extracted by Relion with a box size of 288 pixels and a pixel size of 0.83 Å. Particles were imported into cryoSPARC, and one round of heterogeneous refinement was performed. A total of 183,797 particles were selected. Finally, one round of nonuniform refinement and local refinement was performed, yielding a 3.11 Å map. For data of the PfENT1_Y190A_-Nb19 complex, 2,488,266 particles were picked. A total of 4174 micrographs were selected with 2,292,368 particles. A similar processing procedure was applied. 89,264 particles were selected for final reconstruction. After particles re-extraction, particles were imported into cryoSPARC v.3.3.1. One round of ab inito reconstruction, nonuniform refinement, and local refinement was performed, yielding a 3.34 Å map. Conversions of particle star files between Relion and cryoSPARC were performed mainly using the pyem script^60^. The FSC between the map and model was calculated by Phenix.mtriage^58^.

For PfENT1_GFP_ data, data processing was performed using cryoSPARC. Movies frames were aligned using Patch motion. CTF estimation was performed using Patch CTF. Particles were first picked using a blob picker with partial micrographs. 2D templates were generated by 2D classification. Particle picking of all micrographs was performed by a template picker. A total of 6,684,066 particles from 4508 micrographs were extracted using a box size of 196 pixels and cropped into 98 pixels. After four rounds of 2D classification, 1,801,045 particles were selected. Ab initio reconstruction using partial particles and five rounds of heterogeneous refinement was performed and generated a dataset with 386,720 particles. One round of nonuniform and local refinement was performed. A total about 6,746,245 particles from 4536 micrographs were extracted using a box size of 196 pixels and cropped into 98 pixels. After four rounds of 2D classification, 1,255,179 particles were selected, followed by ab initio reconstruction. Two rounds of heterogeneous refinement were performed using combined selected particle sets, generated a new dataset with 757,172 particles. Two rounds of ab initio reconstruction were performed, generated a new dataset with 598,813 particles. One round of nonuniform and local refinement was performed, followed by particle re-extraction with a box size of 196 pixels, ab initio reconstruction, and new round of nonuniform and local refinement. A 4.37 Å map was obtained. Finally, after three rounds of local refinement with a mask excluded GFP, a 4.15 Å map was obtained. Three rounds of local refinement with a mask excluded GFP generated a 4.15 Å map. 204,716 particles were selected after one round of ab initio reconstruction. Finally, one round of nonuniform generated a 4.04 Å map.

### Model building and refinement

The PfENT1 and Nb48 models were first generated by SWISS-MODEL using the structures of hENT1 (PDB code: 6OB6) and nanobody (PDB code: 3EZJ) as templates, respectively. The two models were then docked into the density map and manually adjusted and rebuilt by COOT^61^. The restraint files of inosine GSK4 were generated by the Phenix.elbow package^62^. The complete model was finally refined in Phenix using real-space refinement with secondary structure and geometry restraints^63^. The atomic model of the PfENT1_Y190A_-Nb19 complex and PfENT1_GFP_ –GSK4 complex is built similarly to that of PfENT1_Y190A_-Nb48. Overfitting of model was diagnosed by refining model using one of the two independent maps from gold-standard refinement, and calculated FSC against both two half maps^64^. The final structure was validated using Molprobity^65^.

### Reporting summary

Further information on research design is available in the Nature Portfolio Reporting Summary linked to this article.

## Supplementary information

Reporting Summary

Supplementary information

## Data Availability

The data that support this study are available from the corresponding authors upon request. Cryo-EM maps and atomic models have been deposited in the Electron Microscopy Data Bank under accession codes: EMD-32618 (PfENT1_Y190A_ in complex with Nb19), EMD-32619 (PfENT1_Y190A_ in complex with Nb48 and inosine), and EMD-33756 (PfENT1_GFP_ in complex with GSK4). Coordinates have been deposited in the Protein Data Bank (PDB) under accession codes: 7WN0 (PfENT1_Y190A_ in complex with Nb19), 7WN1 (PfENT1_Y190A_ in complex with Nb48 and inosine), and 7YDQ (PfENT1_GFP_ in complex with GSK4). Previously solved structures referred in Fig. 4b have been deposited in Protein Data Bank under the accession code: 6OB6 (human equilibrative nucleoside transporter-1). Source data are provided with this paper.

## Source data

Source Data

## References

[1] Organization, W. H. World malaria report 2020. (2021).

[2] Greenwood BM (2008). Malaria: progress, perils, and prospects for eradication. J. Clin. Invest.

[3] Blasco B, Leroy D, Fidock DA (2017). Antimalarial drug resistance: linking Plasmodium falciparum parasite biology to the clinic. Nat. Med.

[4] Menard D, Dondorp A (2017). Antimalarial drug resistance: a threat to malaria elimination. Cold Spring Harb. Perspect. Med..

[5] Ashley EA (2014). Spread of artemisinin resistance in Plasmodium falciparum malaria. N. Engl. J. Med.

[6] Shibeshi MA, Kifle ZD, Atnafie SA (2020). Antimalarial drug resistance and novel targets for antimalarial drug discovery. Infect. Drug Resist.

[7] Su XZ, Lane KD, Xia L, Sa JM, Wellems TE (2019). Plasmodium genomics and genetics: new insights into malaria pathogenesis, drug resistance, epidemiology, and evolution. Clin. Microbiol Rev..

[8] Okombo J, Chibale K (2018). Recent updates in the discovery and development of novel antimalarial drug candidates. Medchemcomm.

[9] Thu AM, Phyo AP, Landier J, Parker DM, Nosten FH (2017). Combating multidrug-resistant Plasmodium falciparum malaria. FEBS J..

[10] Downie MJ, Kirk K, Mamoun CB (2008). Purine salvage pathways in the intraerythrocytic malaria parasite Plasmodium falciparum. Eukaryot. Cell.

[11] Kokina A, Ozolina Z, Liepins J (2019). Purine auxotrophy: Possible applications beyond genetic marker. Yeast.

[12] Carter NS, Landfear SM, Ullman B (2001). Nucleoside transporters of parasitic protozoa. Trends Parasitol..

[13] Landfear SM, Ullman B, Carter NS, Sanchez MA (2004). Nucleoside and nucleobase transporters in parasitic protozoa. Eukaryot. Cell.

[14] de Koning HP, Bridges DJ, Burchmore RJ (2005). Purine and pyrimidine transport in pathogenic protozoa: from biology to therapy. FEMS Microbiol Rev..

[15] Carter NS (2000). Isolation and functional characterization of the PfNT1 nucleoside transporter gene from Plasmodium falciparum. J. Biol. Chem..

[16] Parker MD (2000). Identification of a nucleoside/nucleobase transporter from Plasmodium falciparum, a novel target for anti-malarial chemotherapy. Biochem J..

[17] Rager N, Mamoun CB, Carter NS, Goldberg DE, Ullman B (2001). Localization of the Plasmodium falciparum PfNT1 nucleoside transporter to the parasite plasma membrane. J. Biol. Chem..

[18] Frame IJ, Deniskin R, Arora A, Akabas MH (2015). Purine import into malaria parasites as a target for antimalarial drug development. Ann. NY Acad. Sci..

[19] Cassera MB, Zhang Y, Hazleton KZ, Schramm VL (2011). Purine and pyrimidine pathways as targets in Plasmodium falciparum. Curr. Top. Med Chem..

[20] Zhang M (2018). Uncovering the essential genes of the human malaria parasite Plasmodium falciparum by saturation mutagenesis. Science.

[21] Frame IJ (2015). Yeast-based high-throughput screen identifies Plasmodium falciparum equilibrative nucleoside transporter 1 inhibitors that kill malaria parasites. ACS Chem. Biol..

[22] Sosa Y (2019). Identification via a parallel hit progression strategy of improved small molecule inhibitors of the malaria purine uptake transporter that inhibit plasmodium falciparum parasite proliferation. ACS Infect. Dis..

[23] El Bissati K (2006). The plasma membrane permease PfNT1 is essential for purine salvage in the human malaria parasite Plasmodium falciparum. Proc. Natl. Acad. Sci. USA.

[24] Arora A (2016). Substrate and inhibitor specificity of the plasmodium berghei equilibrative nucleoside transporter type 1. Mol. Pharm..

[25] Meier A, Erler H, Beitz E (2018). Targeting channels and transporters in protozoan parasite infections. Front Chem..

[26] Wright NJ, Lee SY (2021). Toward a molecular basis of cellular nucleoside transport in humans. Chem. Rev..

[27] Downie MJ, Saliba KJ, Howitt SM, Bröer S, Kirk K (2006). Transport of nucleosides across the Plasmodium falciparum parasite plasma membrane has characteristics of PfENT1. Mol. Microbiol.

[28] El Bissati K (2008). Genetic evidence for the essential role of PfNT1 in the transport and utilization of xanthine, guanine, guanosine and adenine by Plasmodium falciparum. Mol. Biochem Parasitol..

[29] Gorman MW, Marble DR, Ogimoto K, Feigl EO (2003). Measurement of adenine nucleotides in plasma. Luminescence.

[30] Simmonds RJ, Harkness RA (1981). High-performance liquid chromatographic methods for base and nucleoside analysis in extracellular fluids and in cells. J. Chromatogr..

[31] Eells JT, Spector R (1983). Purine and pyrimidine base and nucleoside concentrations in human cerebrospinal fluid and plasma. Neurochem Res.

[32] Traut TW (1994). Physiological concentrations of purines and pyrimidines. Mol. Cell Biochem..

[33] Farthing D (2007). An HPLC method for determination of inosine and hypoxanthine in human plasma from healthy volunteers and patients presenting with potential acute cardiac ischemia. J. Chromatogr. B Anal. Technol. Biomed. Life Sci..

[34] Carter NS (2000). Cloning of a novel inosine-guanosine transporter gene from Leishmania donovani by functional rescue of a transport-deficient mutant. J. Biol. Chem..

[35] Huang W, Zeng X, Shi Y, Liu M (2017). Functional characterization of human equilibrative nucleoside transporter 1. Protein Cell.

[36] Wu S (2012). Fabs enable single particle cryoEM studies of small proteins. Structure.

[37] Wright NJ, Lee S-Y (2019). Structures of human ENT1 in complex with adenosine reuptake inhibitors. Nat. Struct. Mol. Biol..

[38] Boswell-Casteel RC, Hays FA (2017). Equilibrative nucleoside transporters-A review. Nucleosides Nucleotides Nucleic Acids.

[39] Drew D, North RA, Nagarathinam K, Tanabe M (2021). Structures and general transport mechanisms by the major facilitator superfamily (MFS). Chem. Rev..

[40] Wang SC (2020). Expansion of the Major Facilitator Superfamily (MFS) to include novel transporters as well as transmembrane-acting enzymes. Biochim Biophys. Acta Biomembr..

[41] Arastu-Kapur S, Ford E, Ullman B, Carter NS (2003). Functional analysis of an inosine-guanosine transporter from Leishmania donovani. The role Conserve. residues, aspartate 389 arginine 393.. J. Biol. Chem..

[42] Visser F (2007). Residues 334 and 338 in transmembrane segment 8 of human equilibrative nucleoside transporter 1 are important determinants of inhibitor sensitivity, protein folding, and catalytic turnover. J. Biol. Chem..

[43] Wang C (2021). Molecular basis for substrate recognition by the bacterial nucleoside transporter NupG. J. Biol. Chem..

[44] Zhou Y (2020). Cryo-EM structure of the human concentrative nucleoside transporter CNT3. PLoS Biol..

[45] Johnson ZL, Cheong CG, Lee SY (2012). Crystal structure of a concentrative nucleoside transporter from Vibrio cholerae at 2.4 A. Nature.

[46] Yan N (2013). Structural advances for the major facilitator superfamily (MFS) transporters. Trends Biochem. Sci..

[47] Quistgaard EM, Low C, Guettou F, Nordlund P (2016). Understanding transport by the major facilitator superfamily (MFS): structures pave the way. Nat. Rev. Mol. Cell Biol..

[48] Yan N (2015). Structural biology of the major facilitator superfamily transporters. Annu Rev. Biophys..

[49] Kaback HR, Smirnova I, Kasho V, Nie Y, Zhou Y (2011). The alternating access transport mechanism in LacY. J. Membr. Biol..

[50] Deng D (2015). Molecular basis of ligand recognition and transport by glucose transporters. Nature.

[51] Wunderlich J (2022). Updated List of Transport Proteins in Plasmodium falciparum. Front Cell Infect. Microbiol.

[52] Gibson DG (2009). Enzymatic assembly of DNA molecules up to several hundred kilobases. Nat. Methods.

[53] Liu, H. et al. Multi-modular engineering of Saccharomyces cerevisiae for high-titre production of tyrosol and salidroside. *Microb Biotechnol*. **14**, 2605–2616, (2021). 10.1111/1751-7915.13667 PMC8601180 32990403

[54] Gietz RD, Schiestl RH (2007). High-efficiency yeast transformation using the LiAc/SS carrier DNA/PEG method. Nat. Protoc..

[55] Mastronarde DN (2003). SerialEM: A program for automated tilt series acquisition on tecnai microscopes using prediction of specimen position. Microsc. Microanal..

[56] Zheng SQ (2017). MotionCor2: anisotropic correction of beam-induced motion for improved cryo-electron microscopy. Nat. Methods.

[57] Rohou A, Grigorieff N (2015). CTFFIND4: Fast and accurate defocus estimation from electron micrographs. J. Struct. Biol..

[58] Jasenko (2018). New tools for automated high-resolution cryo-EM structure determination in RELION-3. eLife.

[59] Punjani A, Rubinstein JL, Fleet DJ, Brubaker MA (2017). cryoSPARC: algorithms for rapid unsupervised cryo-EM structure determination. Nat. Methods.

[60] Daniel Asarnow, E. P. & Cheng, Y. 10.5281/zenodo.3576630 (2019).

[61] Emsley P, Cowtan K (2004). Coot: model-building tools for molecular graphics. Acta Crystallogr. Sect. D. Biol. Crystallogr..

[62] Moriarty NW, Grosse-Kunstleve RW, Adams PD (2009). Electronic ligand builder and optimization workbench (eLBOW): a tool for ligand coordinate and restraint generation. Acta Crystallogr. Sect. D. Biol. Crystallogr..

[63] Adams PD (2010). PHENIX: a comprehensive Python-based system for macromolecular structure solution. Acta Crystallogr D. Biol. Crystallogr.

[64] Amunts A (2014). Structure of the yeast mitochondrial large ribosomal subunit. Science.

[65] Chen VB (2010). MolProbity: all-atom structure validation for macromolecular crystallography. Acta Crystallogr D. Biol. Crystallogr.

