# Supplementary material for: Structural basis of the substrate recognition and inhibition mechanism of Plasmodium falciparum nucleoside transporter PfENT1
Source: Nat Commun. 2023 Mar 28;14:1727. doi: 10.1038/s41467-023-37411-1 (PMC10050424; doi:10.1038/s41467-023-37411-1)
Supplement: Supplementary file 1 — Reporting Summary [file 41467_2023_37411_MOESM1_ESM.pdf]

nature portfolio

Corresponding author(s): Li Guo, Ruobing Ren, and Dong Deng

Last updated by author(s): Mar 10, 2023

Reporting Summary

Nature Portfolio wishes to improve the reproducibility of the work that we publish. This form provides structure for consistency and transparency in reporting. For further information on Nature Portfolio policies, see our Editorial Policies and the Editorial Policy Checklist.

Statistics

For all statistical analyses, confirm that the following items are present in the figure legend, table legend, main text, or Methods section.

n/a	Confirmed	
☐	☒	The exact sample size (*n*) for each experimental group/condition, given as a discrete number and unit of measurement
☐	☒	A statement on whether measurements were taken from distinct samples or whether the same sample was measured repeatedly
☒	☐	The statistical test(s) used AND whether they are one- or two-sided<br>*Only common tests should be described solely by name; describe more complex techniques in the Methods section.*
☒	☐	A description of all covariates tested
☒	☐	A description of any assumptions or corrections, such as tests of normality and adjustment for multiple comparisons
☐	☒	A full description of the statistical parameters including central tendency (e.g. means) or other basic estimates (e.g. regression coefficient) AND variation (e.g. standard deviation) or associated estimates of uncertainty (e.g. confidence intervals)
☒	☐	For null hypothesis testing, the test statistic (e.g. *F*, *t*, *r*) with confidence intervals, effect sizes, degrees of freedom and *P* value noted<br>*Give P values as exact values whenever suitable.*
☒	☐	For Bayesian analysis, information on the choice of priors and Markov chain Monte Carlo settings
☒	☐	For hierarchical and complex designs, identification of the appropriate level for tests and full reporting of outcomes
☒	☐	Estimates of effect sizes (e.g. Cohen's *d*, Pearson's *r*), indicating how they were calculated

Our web collection on statistics for biologists contains articles on many of the points above.

Software and code

Policy information about availability of computer code

Data collection	SerialEM 3.7.10,EPU 2.10.0
Data analysis	GraphPad Prism 8 software (version 7.0.0), PEAQ-ITC analysis software (MicroCal, version 1.1.0.1062), cryoSPARC v.2.14.2, cryoSPARC v.3.3.1, AlphaFold2, COOT 0.9.6, PHENIX 1.20.1, UCSF Chimera 1.14, Relion3.1, Pymol v2.3.5.Molprobity(integrated in Phenix 1.20.1-4487-000)

For manuscripts utilizing custom algorithms or software that are central to the research but not yet described in published literature, software must be made available to editors and reviewers. We strongly encourage code deposition in a community repository (e.g. GitHub). See the Nature Portfolio guidelines for submitting code & software for further information.

Data

Policy information about availability of data

All manuscripts must include a data availability statement. This statement should provide the following information, where applicable:

- Accession codes, unique identifiers, or web links for publicly available datasets
- A description of any restrictions on data availability
- For clinical datasets or third party data, please ensure that the statement adheres to our policy

Cryo-EM maps and atomic models have been deposited in the Electron Microscopy Data Bank under accession codes: EMD-32618 (PfNT1Y190A in complex with NB19), EMD-32619 (PfNT1Y190A in complex with NB48 and inosine), and EMD-33756 (PfNT1GFP in complex with GSK4) and in the Protein Data Bank under

accession codes: 7WN0 PfNT1Y190A in complex with NB19), 7WN1 (PfNT1Y190A in complex with NB48 and inosine), and 7YDQ (PfNT1GFP in complex with GSK4). Previously solved structures referred in figure 4b have been deposited in Protein Data Bank under the accession code: 6OB6 (human equilibrative nucleoside transporter-1)

Human research participants

Policy information about studies involving human research participants and Sex and Gender in Research.

Reporting on sex and gender	not applicable
Population characteristics	not applicable
Recruitment	not applicable
Ethics oversight	not applicable

Note that full information on the approval of the study protocol must also be provided in the manuscript.

Field-specific reporting

Please select the one below that is the best fit for your research. If you are not sure, read the appropriate sections before making your selection.

☒ Life sciences ☐ Behavioural & social sciences ☐ Ecological, evolutionary & environmental sciences

For a reference copy of the document with all sections, see nature.com/documents/nr-reporting-summary-flat.pdf

Life sciences study design

All studies must disclose on these points even when the disclosure is negative.

Sample size	All the samples prepared for ITC experiments and transporter assay experiments were performed at least three times, which provided reproducible results. For cryoEM analysis, density maps were calculated from hundreds of thousands of particle images (Supplementary Table 1). No statistic methods was used to predetermine sample size.
Data exclusions	NO data was systematically excluded. The procedure of generating cryo-EM maps involves sorting out the particles that are false-positively piced, damaged or represent minor conformations. The procedure is part of the cryoSPARC v.3.3.1 and cryoSPARC v.2.1.42 pipeline.
Replication	All experiments in this work including ITC , transporter assay and microscopic imaging, were performed at least three times. All attempts at replication were successful.
Randomization	Randomization is not applicable for the experiments in this study, as single particle analysis is automatically processed by software packages.
Blinding	Blinding is not applicable for the experiments in this study, because it is not technically or practically feasible to do so for either the cryo-EM structure determination or the function assays.

Reporting for specific materials, systems and methods

We require information from authors about some types of materials, experimental systems and methods used in many studies. Here, indicate whether each material, system or method listed is relevant to your study. If you are not sure if a list item applies to your research, read the appropriate section before selecting a response.

Materials & experimental systems

n/a	Involved in the study	
☐	☒	Antibodies
☐	☒	Eukaryotic cell lines
☒	☐	Palaeontology and archaeology
☒	☐	Animals and other organisms
☒	☐	Clinical data
☒	☐	Dual use research of concern

Methods

n/a	Involved in the study	
☒	☐	ChIP-seq
☒	☐	Flow cytometry
☒	☐	MRI-based neuroimaging

Antibodies

Antibodies used	Rabbit anti-HA Tag pAb , ABclonal, catalogue number AE036, 1:4000 dilution<br>Goat anti-Rabbit IgG H&L HRP, ZenBio, catalogue number 511203, 1:4000 dilution
Validation	All antibodies used were validated by the commercial vendors:<br>Anti-HA:https://abclonal.com.cn/catalog/AE036<br>Goat anti-Rabiit igG:http://www.zen-bio.cn/prod_view.aspx?IsActiveTarget=True&TypeId=145&Id=507322&FId=t3:145:3

Eukaryotic cell lines

Policy information about cell lines and Sex and Gender in Research

Cell line source(s)	Sf9 cells were purchased from Thermo Fischer Scientific (cat # 11496015); Saccharomyces cerevisiae cells purchased from ATCC(cat # ATCC 201388)
Authentication	None of the cell lines used were authentication.
Mycoplasma contamination	The cell lines listed above were not tested for mycoplasma contamination.
Commonly misidentified lines<br>(See ICLAC register)	none.
