# Supplementary material for: Structural basis of the substrate recognition and inhibition mechanism of Plasmodium falciparum nucleoside transporter PfENT1
Source: Nat Commun. 2023 Mar 28;14:1727. doi: 10.1038/s41467-023-37411-1 (PMC10050424; doi:10.1038/s41467-023-37411-1)
Supplement: Supplementary file 2 — Supplementary information [file 41467_2023_37411_MOESM2_ESM.pdf]

Structural basis of the substrate recognition and inhibition mechanism of *Plasmodium falciparum* nucleoside transporter PfENT1

Supplementary information

Supplementary Table 1: Cryo-EM data collection and refinement statistics

Supplementary Figures 1-15

Supplementary Table 1. Cryo-EM data collection and refinement statistics

	Apo<br>EMDB-32618<br>PDB: 7WN0	Inosine complex<br>EMDB-32619<br>PDB: 7WN1	GSK4 complex<br>EMDB-33756<br>PDB: 7YDQ
**Data collection and processing**			
Magnification	105,000	105,000	130,000
Voltage (kV)	300	300	300
Electron exposure (e–/Å^2)	57.5	56.3	52.452
Defocus range (μm)	-1.2 - -1.8	-1.0 - -1.8	-1.1 - -1.8
Pixel size (Å)	0.83	0.83	1.1
Symmetry imposed	C1	C1	C1
Initial particle images (no.)	2,488,266	4,651,677	13,430,311
Final particle images (no.)	89,264	183,797	204,716
Map resolution (Å)	3.34	3.11	4.04
FSC threshold	0.143	0.143	0.143
Map resolution range (Å)	3.0-4.5	2.6-4.6	3.5-4.5
**Refinement**			
Initial model used (PDB code)	7WN1, 3EZJ	6OB6, 3EZJ	7WN0
Model resolution (Å)	3.3/3.6	3.0/3.2	3.9/4.2
FSC threshold	0.143/0.5	0.143/0.5	0.143/0.5
Model composition			
Nonhydrogen atoms	3879	3901	2964
Protein residues	494	493	369
Ligands	0	1	1
B factors (Å^2)			
Protein	119.76	65.41	61.33

Ligand	0	59.93	36.57
R.m.s. deviations			
Bond lengths (Å)	0.003	0.007	0.005
Bond angles (°)	0.572	0.648	0.931
Validation			
MolProbity score	1.70	1.67	1.80
Clashscore	9.34	7.77	7.67
Rotamer outliers (%)	0.00	0.00	0.91
Ramachandran plot			
Favored (%)	96.72	96.3	94.49
Allowed (%)	3.28	3.7	5.51
Disallowed (%)	0	0	0
Model vs. map CC			
CC (mask)	0.82	0.87	0.73
CC (box)	0.58	0.64	0.55
CC (peaks)	0.49	0.56	0.49
CC (volume)	0.81	0.86	0.68
Mean CC for ligands	--	0.81	0.80

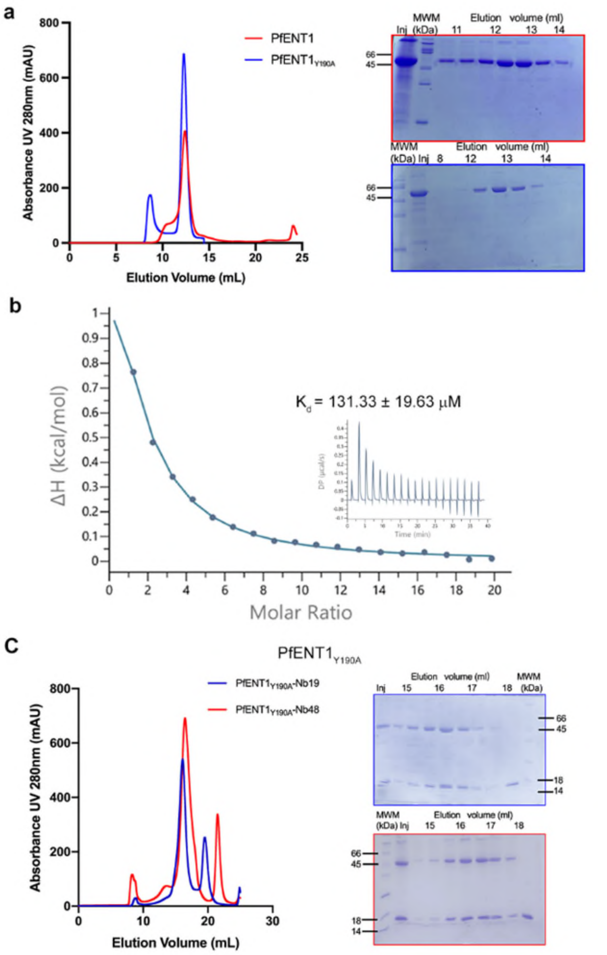

Supplementary Fig. 1 . Characterization of wild-type PfENT1 and PfENT1$_{Y190A}$.

a. Purification of wild-type PfENT1 and PfENT1$_{Y190A}$ in size-exclusion chromatography (Superdex 200 10/300 Increase). The wild-type PfENT1 and PfENT1$_{Y190A}$ were purified in buffer containing 25 mM MES pH 6.0, 150 mM NaCl, and 0.06% cymal6. The peak fractions were employed in SDS–PAGE. **b.** Inosine binding of PfENT1$_{Y190A}$ measured by ITC. **c.** Reconstitution of the PfENT1/nanobody complex in size-exclusion chromatography. A representative size-exclusion chromatography chromatogram of PfENT1$_{Y190A}$-Nb19 and PfENT1$_{Y190A}$-Nb48 complex (Superose 6 10/300 Increase). The purification buffer contained 25 mM MES pH 6.0, 150 mM NaCl, and 0.06% GDN. SDS–PAGE was used to detect the protein peak fractions, which were stained with Coomassie blue. Source data are provided as a Source Data file.

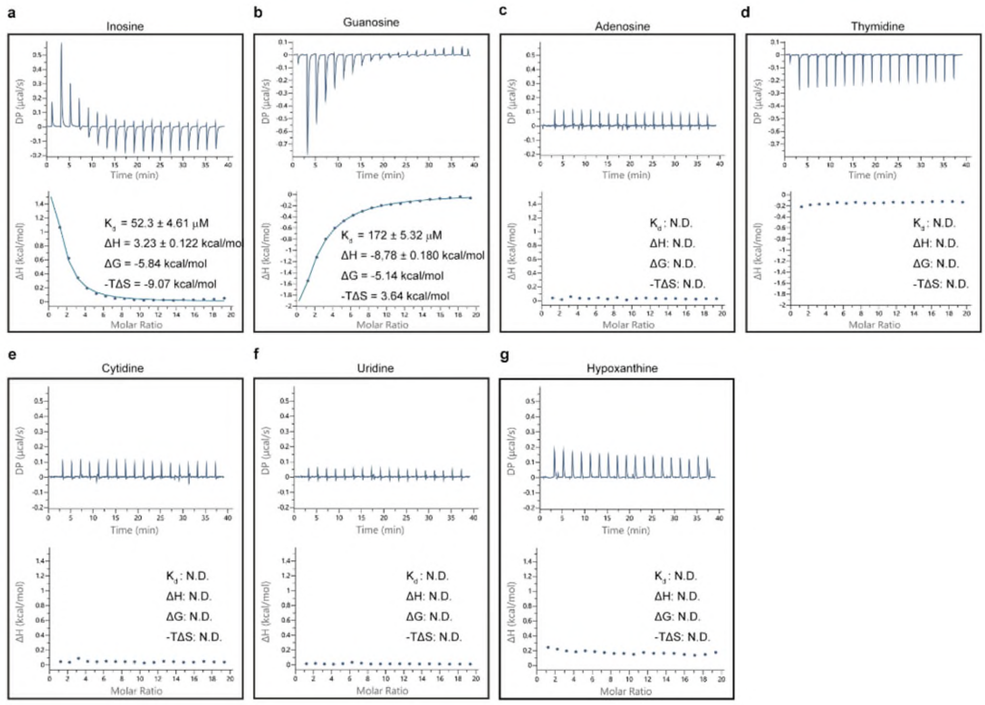

Supplementary Fig. 2 ITC binding data and analyses a–g. Differential power traces and heats of injection traces of the specified nucleoside in the syringe of the ITC instrument. The error bars were obtained from a fit of the data points of the particular ITC experiments. The experimental conditions are described in the Methods section.

a

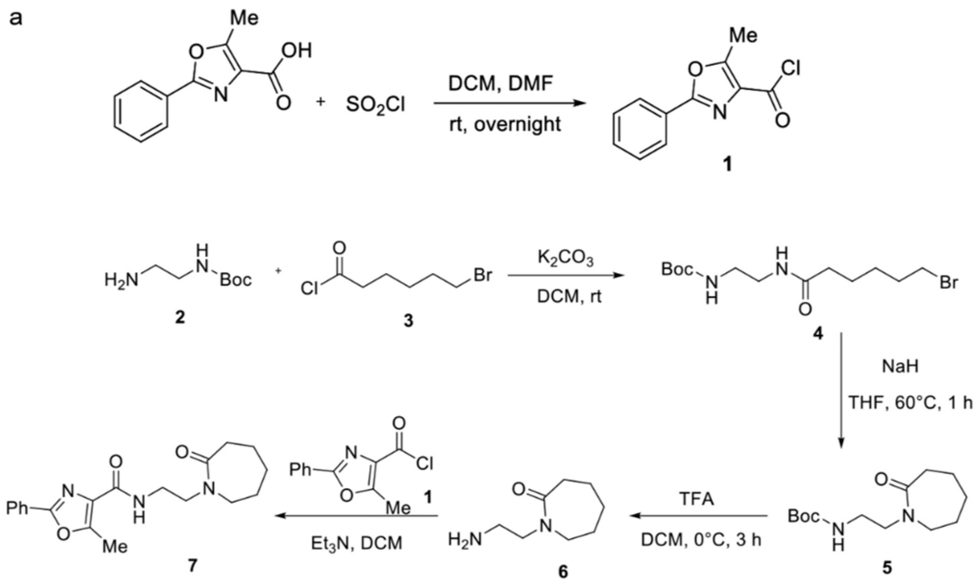

b

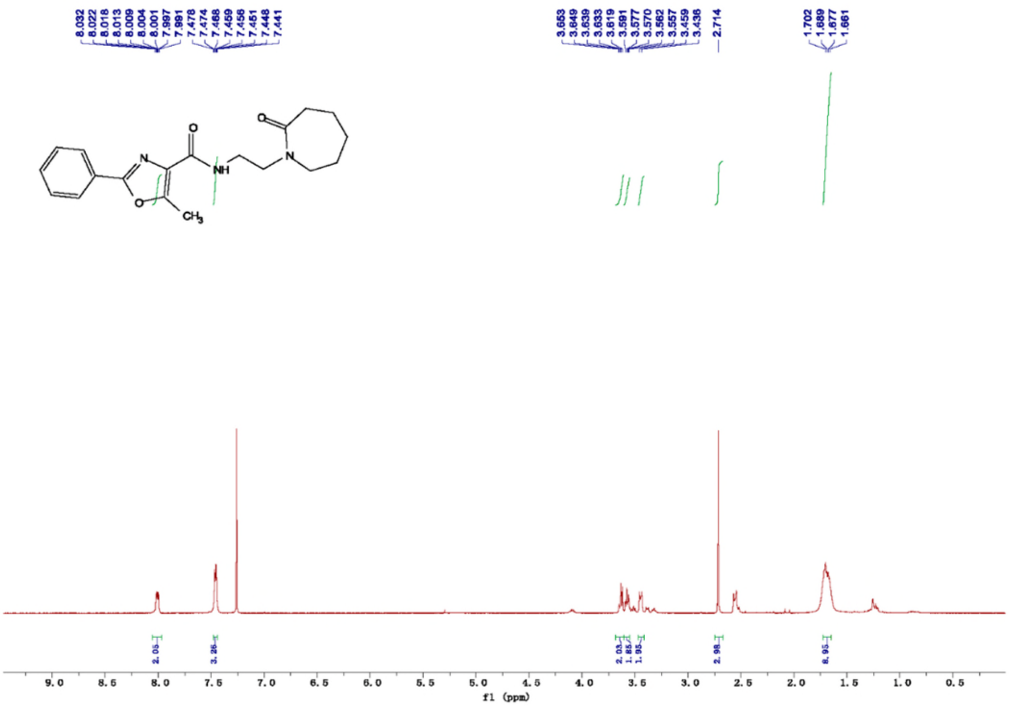

Supplementary Fig. 3. Synthesis of GSK4 a. Seven step chemical synthesis of the GSK4. **b.** NMR spectroscopy of the GSK4.

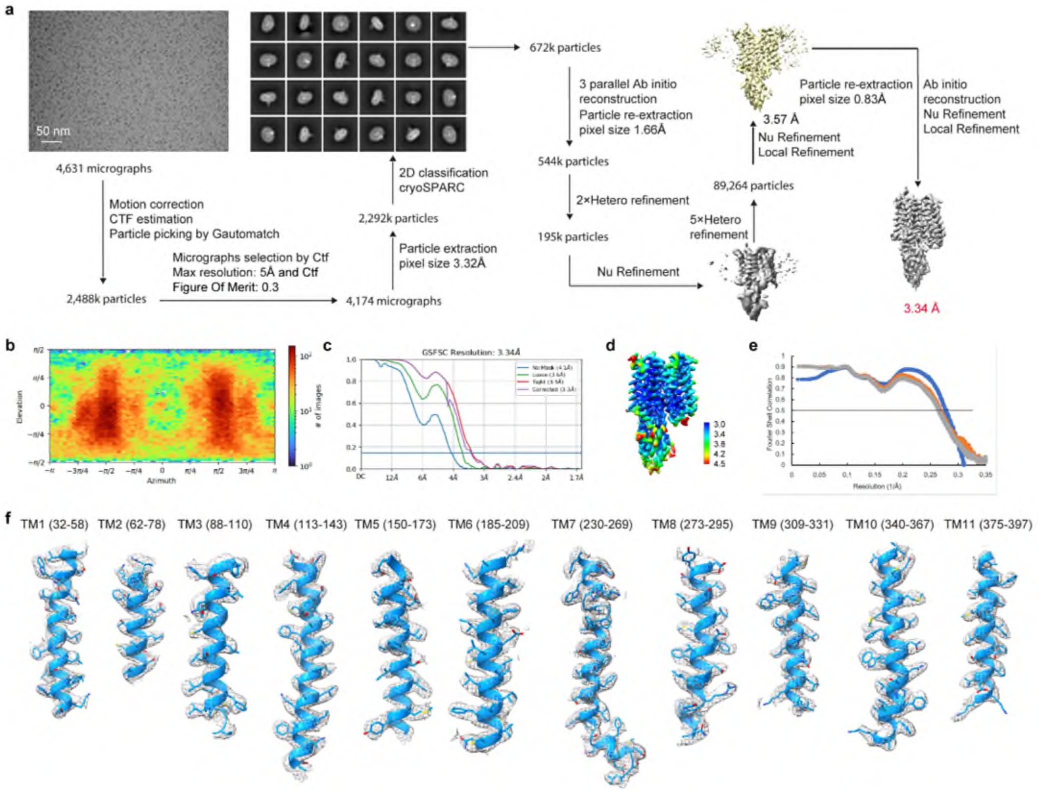

Supplementary Fig. 4. Structural determination of PfENT1-Nb19. a. Flowchart for EM data processing of apo PfENT1-Nb19. Details can be found in the Methods. **b.** Angular distribution of the particles used for the final reconstructions. **c.** Fourier shell correlation (FSC) curves of PfENT1-Nb19. **d.** Local resolution of PfENT1-Nb19 estimated by cryoSPARC. **e.** Fourier shell correlation (FSC) between map and model of PfENT1-Nb19. FSC curve of the final refined model against the full map of PfENT1-Nb19, colored in blue. FSC curve of the model refined against the first half map against the same map, colored in orange. FSC curve of the model refined against the first half map against the second half map, colored in gray. **f.** The density maps of the transmembrane helixes of PfENT1 in PfENT1-Nb19 are shown as mesh.

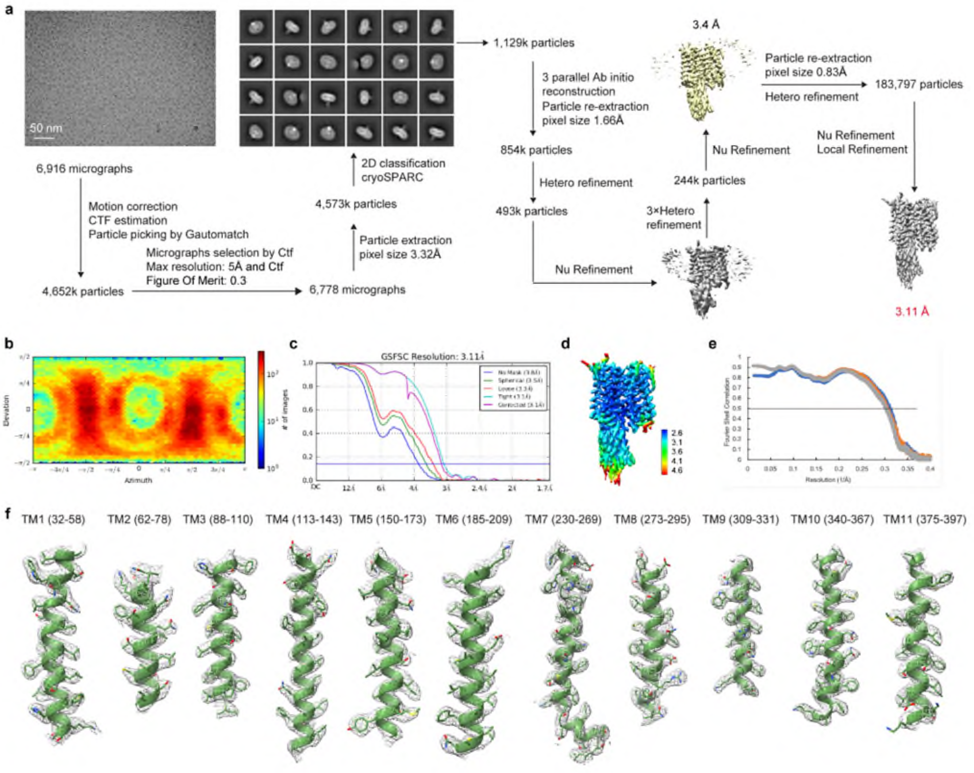

Supplementary Fig. 5. Structural determination of PfENT1-Nb48. a. Flowchart for EM data processing of PfENT1-Nb48. Details can be found in the Methods. **b.** Angular distribution of the particles used for the final reconstructions. **c.** Fourier shell correlation (FSC) curves of PfENT1-Nb48. **d.** Local resolution of PfENT1-Nb48 estimated by cryoSPARC. **e.** Fourier shell correlation (FSC) between map and model of PfENT1-Nb48. FSC curve of the final refined model against the full map of PfENT1-Nb48, colored in blue. FSC curve of the model refined against the first half map against the same map, colored in orange. FSC curve of the model refined against the first half map against the second half map, colored in gray. **f.** The density maps of the transmembrane helixes of PfENT1 in PfENT1-Nb48 are shown as mesh.

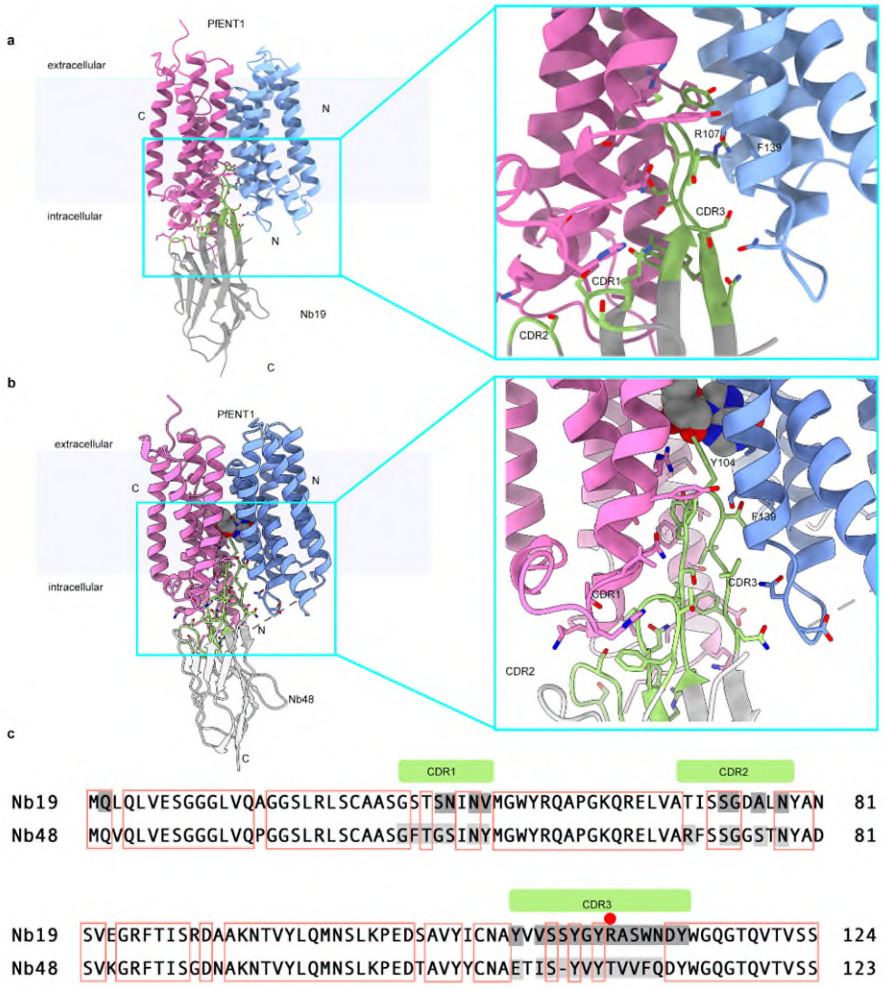

Supplementary Fig. 6. Interfaces between PfENT1$_{Y190A}$ and nanobodies. **a.** The interface between PfENT1$_{Y190A}$ and Nb19. **b.** The interface between PfENT1$_{Y190A}$ and Nb48. **c.** Sequence alignment of Nb19 and Nb48. The highly conserved residues are labeled by the red box. The residues of Nb19 and Nb48 interacting with PfENT1$_{Y190A}$ are shown in dark gray and light gray, respectively. The red circle represents the arginine residue of Nb19, which interacts with PfENT1$_{Y190A}$ and blocks the binding of inosine.

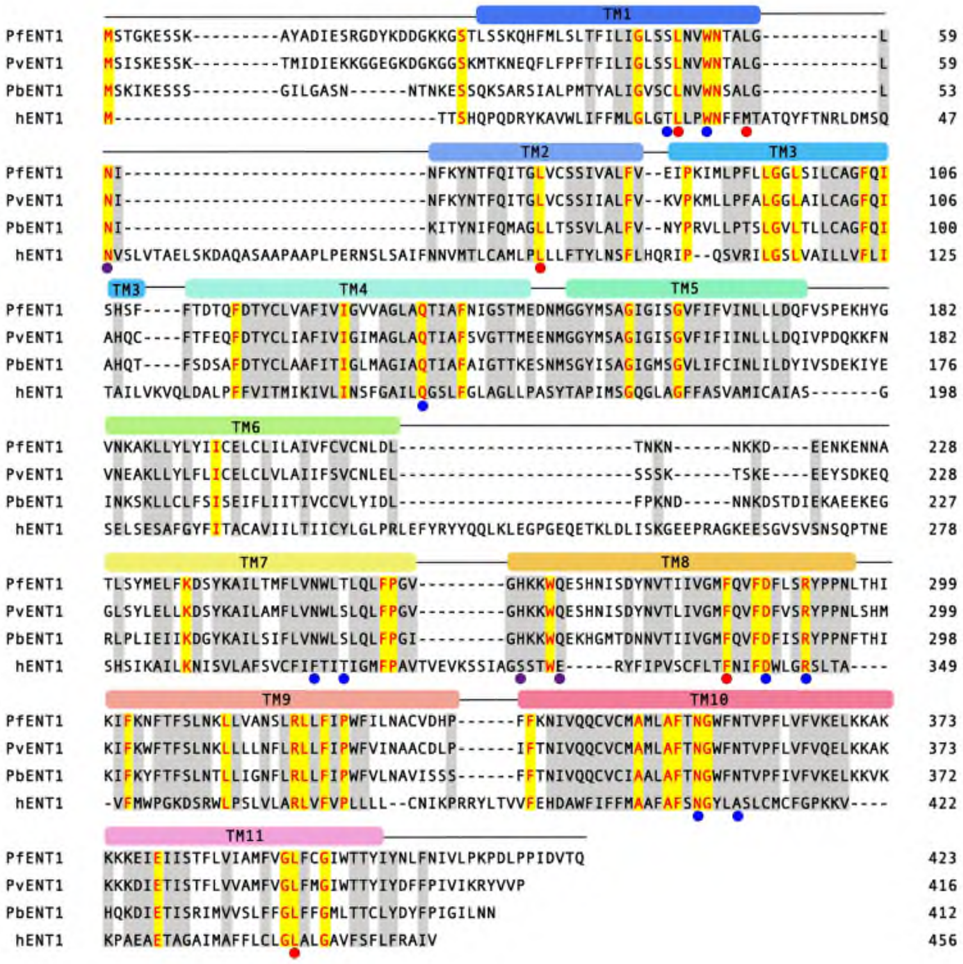

Supplementary Fig. 7. Sequence alignment of ENTs. PfENT1 (UniProt no. Q9NIH9), PvENT1 (UniProt no. A0A564ZY70), PbENT1 (UniProt no. A0A113SRM1), and hENT1 (UniProt no. Q99808). Sequences were aligned with Clustal W. Highly conserved residues and variant residues are shaded yellow and gray. Residues from polar and hydrophobic responsible for substrate binding are indicated by blue and red circles under the sequences, respectively. Residues responsible for alternating access are indicated by purple circles. PfENT1 shares sequence identities of 74%, 58%, and 15% with PvENT1, PbENT1, and hENT1, respectively.

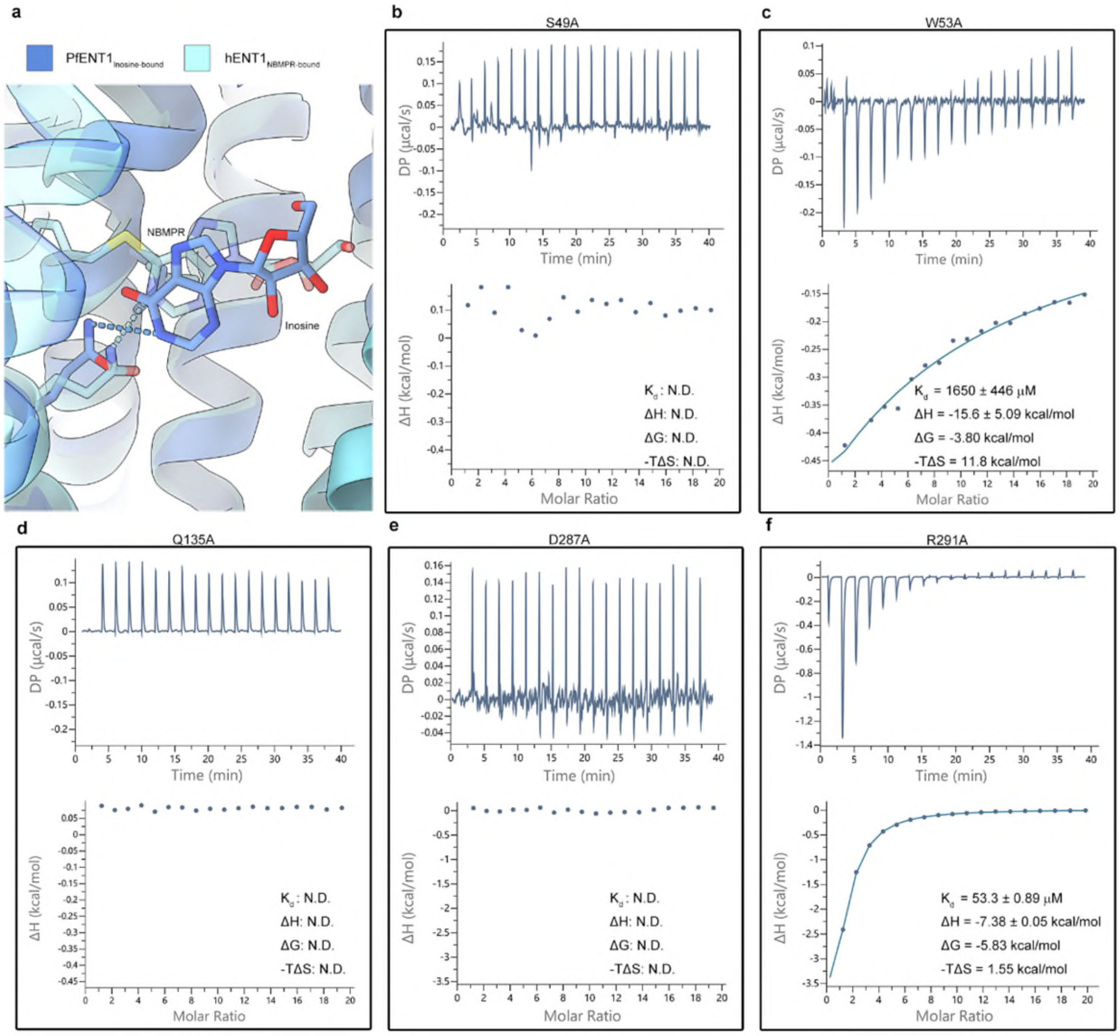

Supplementary Fig. 8. ITC binding data and analyses between PfENT1 mutants and inosine a. The superposition of PfENT1$_{\text{Inosine-bound}}$ and hENT1$_{\text{NBMPR-bound.}}$ **b–f.** Differential power traces and heats of injection traces of the specified PfENT1 mutant proteins in the cell of the ITC instrument. The error bars were obtained from a fit of the data points of the particular ITC experiments. The experimental conditions are described in the Methods section.

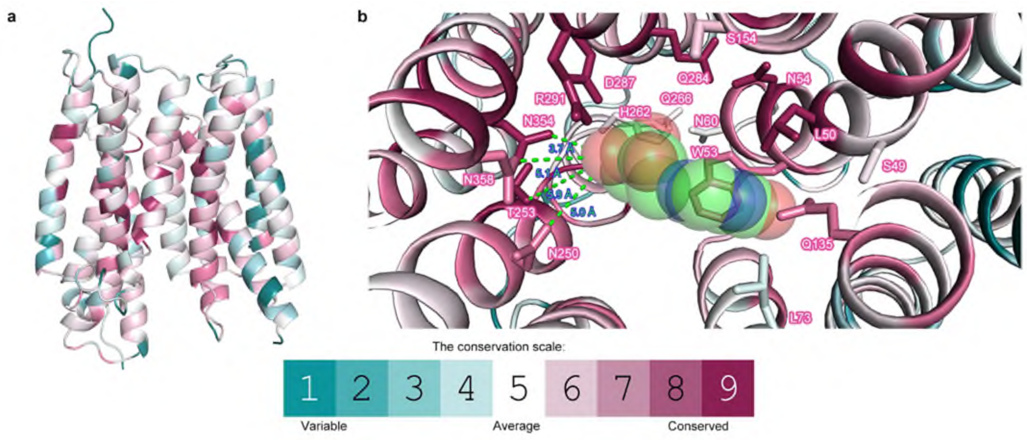

Supplementary Fig. 9. **ConSurf evolutionary conservation analysis of proteins in the ENT family. a,b** Analysis was carried out automatically. Sequence identity was defined between 25% and 80%. Eighty-seven homologs of PfENT1 were used for the analysis. PfENT1 is shown as a ribbon, and the key residues are shown as sticks. The structure is colored by a score of ConSurf evolutionary conservation analysis.

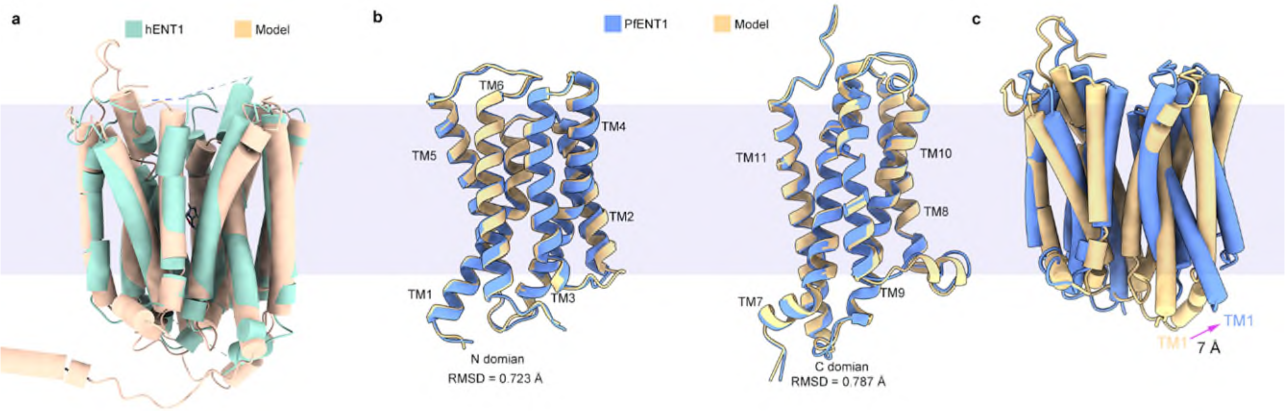

Supplementary Fig. 10. Superposition of PfENT1, hENT1 and the predicted model. a. Superposition of the predicted model of PfENT1 and hENT1 **b.** Superposition of the N and C domains of PfENT1 and the model. **c.** Superposition of the predicted model of PfENT1 and inosine-bound PfENT1 complex.

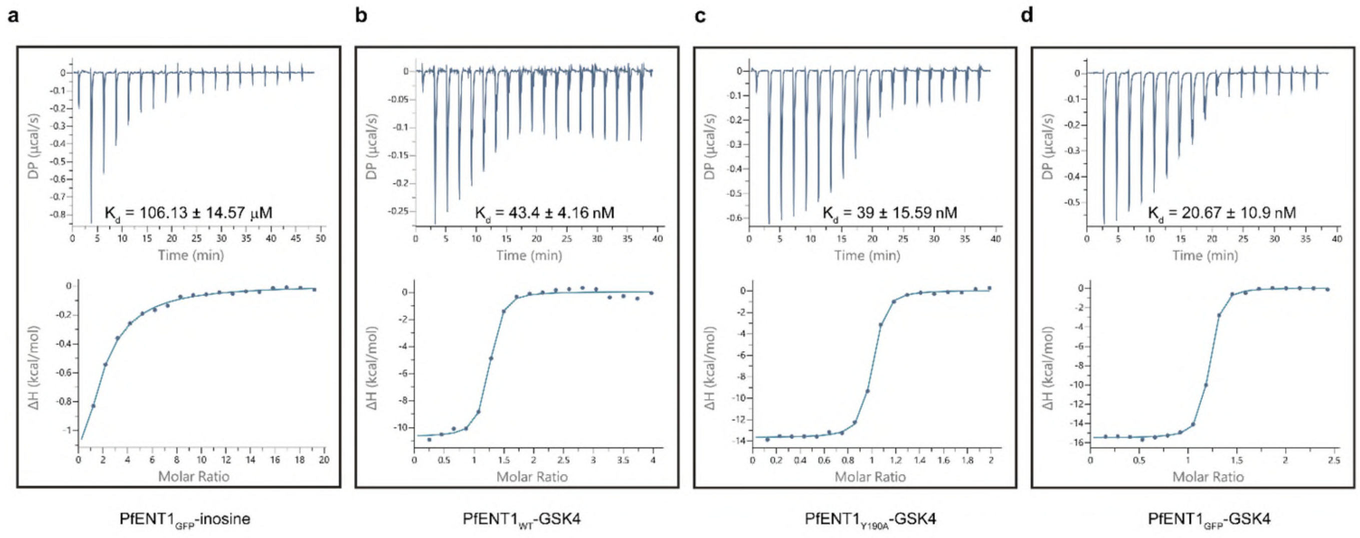

Supplementary Fig. 11. ITC binding data and analyses a–d. Differential power traces and heats of injection traces of the specified samples in the syringe and cell of the ITC instrument. The binding affinity (K_d) is presented as the value of the mean±SD (n=3), n means independent experiment. The experimental conditions are described in the Methods section.

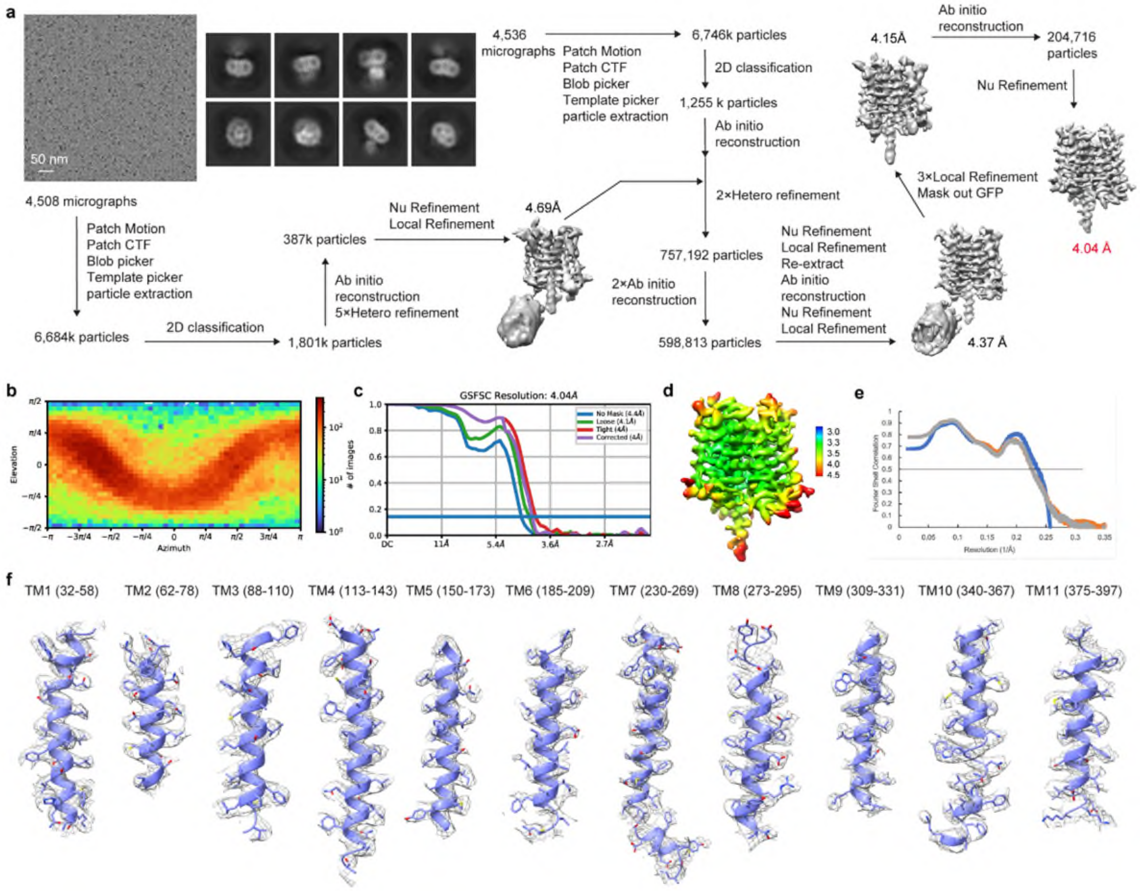

Supplementary Fig. 12. Structural determination of PfENT1$_{GFP}$ a. Flowchart for EM data processing of PfENT1$_{GFP}$. Details can be found in the Methods. **b.** Angular distribution of the particles used for the final reconstructions. **c.** Fourier shell correlation (FSC) curves of PfENT1$_{GFP}$. **d.** Local resolution of PfENT1$_{GFP}$ estimated by cryoSPARC. **e.** Fourier shell correlation (FSC) between map and model of PfENT1$_{GFP}$. FSC curve of the final refined model against the full map of PfENT1$_{GFP}$, colored in blue. FSC curve of the model refined against the first half map against the same map, colored in orange. FSC curve of the model refined against the first half map against the second half map, colored in gray. **f.** The density maps of the transmembrane helixes of PfENT1 in PfENT1$_{GFP}$ are shown as mesh.

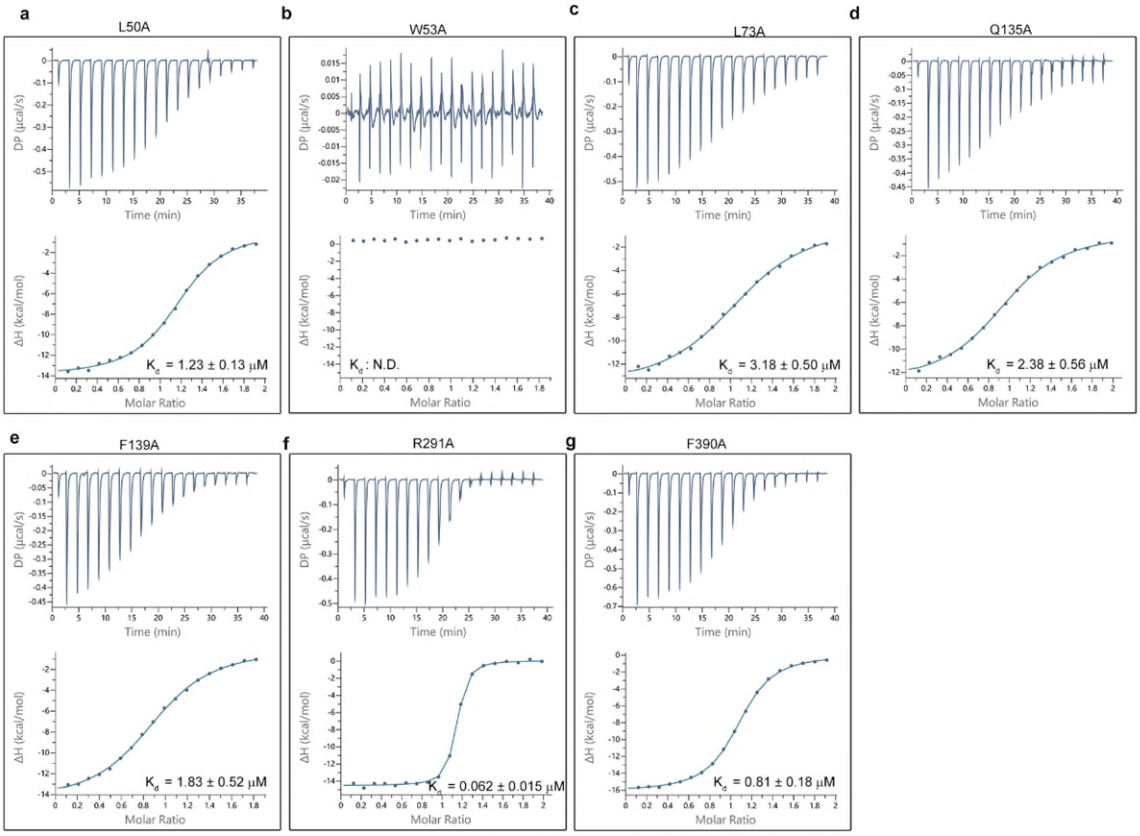

Supplementary Fig. 13. ITC binding data and analyses between PfENT1 mutants and GSK4 a–g. Differential power traces and heats of injection traces of the specified PfENT1 mutant proteins in the cell of the ITC instrument. The experimental conditions are described in the Methods section

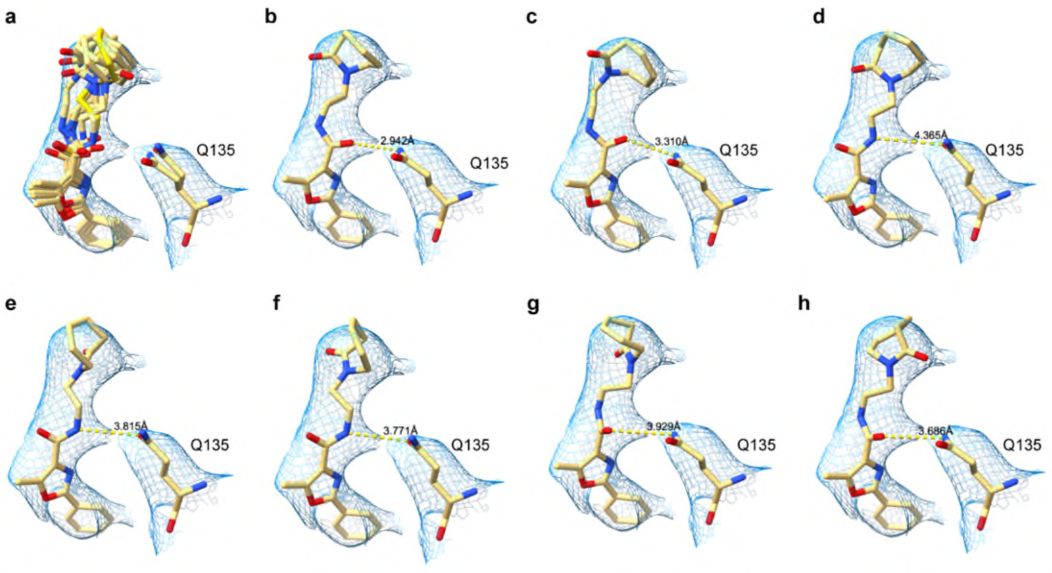

i

Clash scores and local cross-correlation of the ligand to the density of PfENT1 structures with different GSK4 binding poses

	GSK4 Pose b	GSK4 Pose c	GSK4 Pose d	GSK4 Pose e	GSK4 Pose f	GSK4 Pose g	GSK4 Pose h
Clashscore	7.67	8.01	7.84	7.84	7.84	7.67	7.84
Mean CC for ligands	0.80	0.78	0.79	0.81	0.81	0.80	0.80

Supplementary Fig. 14. Binding poses of GSK4 a. Alignment of seven binding poses of GSK4. GSK4 and Q135 are shown in sticks and fitted in density maps (blue mash).**b.** Selected GSK4 binding poses. Pose b was selected as the most rational binding pose and is shown in figure 5. GSK4 and Q135 are shown in sticks and fitted in density maps (blue mash). Atoms are colored: carbon (yellow), oxygen (red), and nitrogen (blue). Interaction between GSK4 and Q135 is showed by dashed line. **c-h.** Another possible GSK4 binding poses. GSK4 and Q135 are shown as same as b. **i.** Summary of clash scores and local cross-correlation of the ligand to the density of PfENT1 structures with different GSK4 binding poses

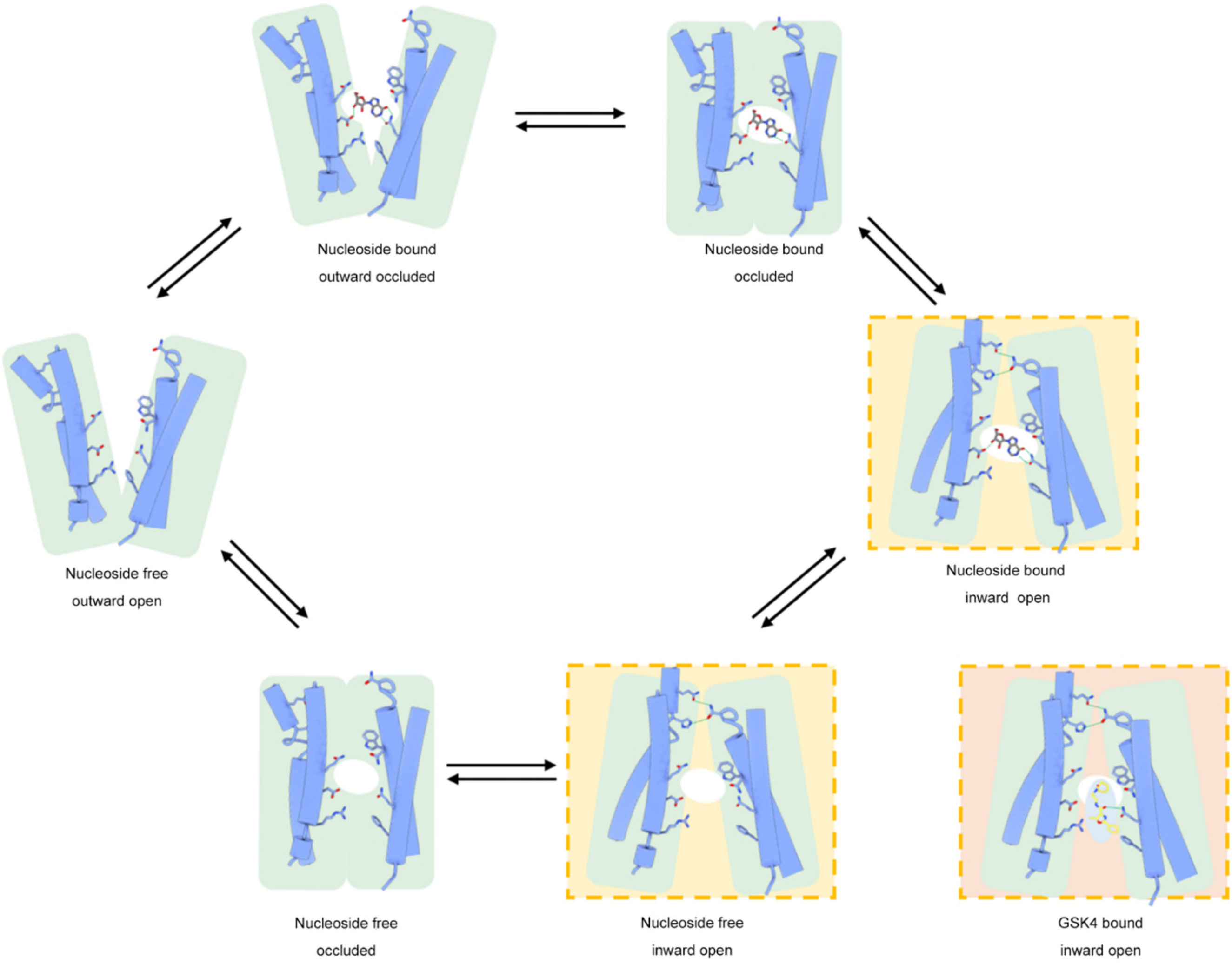

Supplementary Fig. 15. The alternating access model of ENTs. Alternating access of PfENT1 is achieved through the rocker switch.
