# Supplementary figures and images for: Structural basis of the substrate recognition and inhibition mechanism of Plasmodium falciparum nucleoside transporter PfENT1
Source: Nat Commun. 2023 Mar 28;14:1727. doi: 10.1038/s41467-023-37411-1 (PMC10050424; doi:10.1038/s41467-023-37411-1)

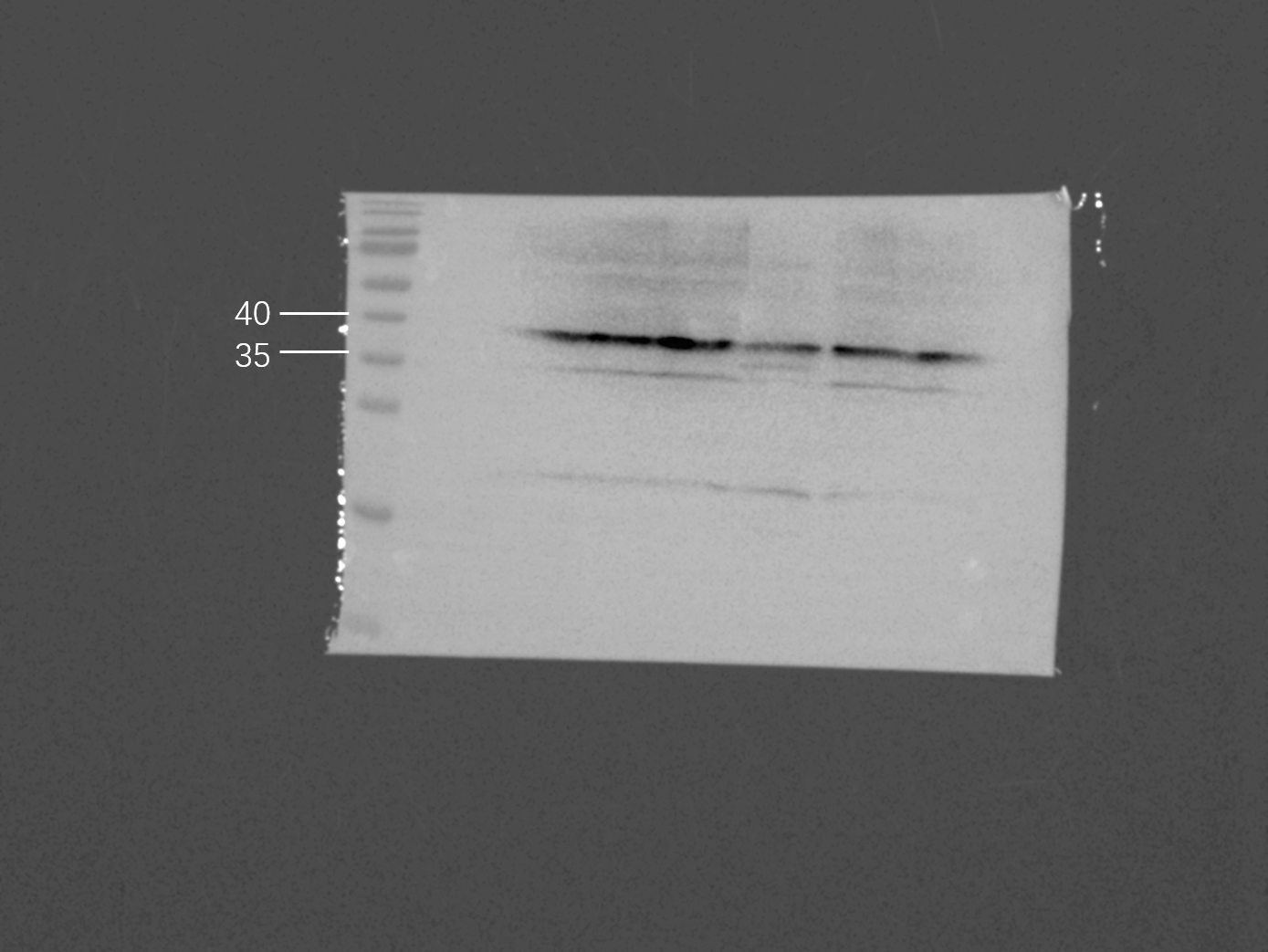

Supplement: Supplementary file 3 — Source Data [file 41467_2023_37411_MOESM3_ESM.zip › Source Data file/Figure 3e.tif]
